# Pathogen patterns in viral ARDS on V-V ECMO: a single-center experience

**DOI:** 10.1007/s15010-026-02879-8

**Published:** 2026-07-15

**Authors:** Jannis Krais, Matthias Lubnow, Alois Philipp, Maik Foltan, Florian Geismann, Christoph Fisser, Florian Hitzenbichler, Bernd Salzberger, Stilla Bauernfeind, Barbara Schmid, Dirk Lunz, Johannes Steinmann, Christoph Eissnert, Martin Kieninger, Walter Petermichl, Roland Schneckenpointner, Alexander Dietl, Lars S. Maier, Bernhard Graf, Thomas Müller, Clemens Wiest

**Affiliations:** 1 Department of Internal Medicine II/Cardiology and Pulmonology, University Hospital Regensburg, Regensburg, Germany; 2 Department of Cardiothoracic Surgery, University Hospital Regensburg, Regensburg, Germany; 3 Department of Pulmonology, Caritas Hospital St. Maria, Donaustauf, Germany; 4 Department of Infectiology, University Hospital Regensburg, Regensburg, Germany; 5 Institute for Microbiology and Hygiene, University Hospital Regensburg, Regensburg, Germany; 6 Department of Anesthesiology and Intensive Care, University Hospital Regensburg, Regensburg, Germany; 7 Department of Cardiology, Angiology and Intensive Care Medicine, Deutsches Herzzentrum der Charité (DHZC), Campus Benjamin Franklin, Hindenburgdamm 30, 12203 Berlin, Germany; 8 Department of Pulmonology and Conservative Intensive Care Medicine, Barmherzige Brueder Hospital Regensburg, Regensburg, Germany

**Keywords:** Extracorporeal membrane oxygenation (ECMO), Acute respiratory distress syndrome (ARDS), COVID-19, Influenza, Superinfection, Pulmonary aspergillosis

## Abstract

**Background:**

Viral acute respiratory distress syndrome (ARDS) is a common cause of respiratory failure requiring veno-venous extracorporeal membrane oxygenation (V-V ECMO). Pulmonary bacterial superinfections, pulmonary aspergillosis, and viral infections or reactivations frequently occur, but data on their incidence, timing, and microbiological spectrum remain limited. We analyzed infectious complications in a large cohort of V-V ECMO-treated patients with viral ARDS.

**Methods:**

We retrospectively evaluated all patients treated at University Hospital Regensburg (UKR) requiring V-V ECMO for COVID-19 (March 2020–May 2022) or influenza (May 2012–December 2022).

**Results:**

We included 147 COVID-19 patients (median age 55.3 years; 77% male; SOFA score 9; V-V ECMO duration 23 days) and 72 influenza patients (median age 55.3 years; 53% male; SOFA score 13; V-V ECMO duration 16 days). COVID-19 patients showed higher cumulative incidences of pulmonary bacterial superinfection (61% vs 35%), aspergillosis (33% vs. 22%), CMV reactivation (18%vs. 4%) and HSV reactivation (49% vs. 26%), however incidence-density rates were comparable between groups with incidence rate ratios (IRR) of 1.10, 0.93, 1.36 and 1.12, respectively. First bacterial pulmonary infection occurred a median of 2 days after V-V ECMO initiation in COVID-19 and 1 day before initiation in influenza patients. Predominant bacterial pathogens were Klebsiella spp. (28%), Staphylococcus aureus (27%) and Escherichia coli (11%) in COVID-19, and Staphylococcus aureus (25%) and Streptococcus pneumoniae (25%) in influenza.

**Conclusion:**

Patients with COVID-19 requiring V-V ECMO showed a distinct trajectory of infectious complications compared to Influenza, particularly bacterial, HSV, CMV, and fungal infections, highlighting their infectious burden.

**Supplementary Information:**

The online version contains supplementary material available at https://doi.org/10.1007/s15010-026-02879-8.

## Introduction

Viral respiratory infections occur in recurrent waves, with the most recent global crisis being the COVID-19 pandemic. The previous influenza pandemic occurred in 2009. Globally, COVID-19 has caused over 6 million deaths [1], with thousands of patients requiring mechanical ventilation and veno-venous extracorporeal membrane oxygenation (V-V ECMO) [2–4]. Patients receiving mechanical ventilation are at high risk of bacterial superinfection, including ventilator-associated pneumonia (VAP) [5]; however, data on patients with viral ARDS requiring V-V ECMO remain limited [6–9]. Reported VAP incidence in COVID-19 patients on V-V ECMO is as high as 86%. Most VAP cases are caused by Enterobacteriaceae (70%) [8], with a high prevalence (57%) of extended spectrum beta-lactamase (ESBL) producers. In contrast, studies excluding V-V ECMO patients, report predominantly Gram-positive pathogens [10].

COVID-19 associated pulmonary Aspergillosis (CAPA) occurs in up to 27.7% of cases [11]. Viral reactivations are also common, with herpes simplex virus (HSV) reported in 31% [12] and cytomegalovirus (CMV) in 9–15% [13, 14] of patients. These complications are associated with increased mortality, yet detailed data on patients supported with V-V ECMO are scarce [6, 12, 14].

This study aimed to characterize and compare the prevalence, timing and microbiological spectrum of superinfections in viral ARDS caused by SARS-CoV2 or influenza in a large cohort of V-V ECMO-treated patients.

## Methods

### Study design

All consecutive ICU patients at the University Hospital Regensburg (UKR) with confirmed COVID-19 pneumonia (March 2020–May 2022) or influenza pneumonia (May 2012–December 2022) requiring V-V ECMO were included. Clinical, microbiological, and outcome data were retrospectively extracted from electronic medical records, ICU documentation systems and institutional microbiology databases. The UKR is a tertiary referral hospital providing approximately 200 ECMO treatments annually. This retrospective cohort study was reported in accordance with the STROBE statement. The study protocol was approved by the local Ethics Committee of the University Hospital Regensburg (approval number 23-3368-104). Owing to the retrospective nature of the study, the requirement for written informed consent was waived.

### Microbiological testing

All ventilated COVID-19 patients receiving V-V ECMO underwent fiberoptic bronchoscopy with bronchoalveolar lavage (BAL) for microbiological diagnostics. BAL was obtained from lung regions with the most purulent secretions or pronounced ground-glass opacities and performed (1) at ICU admissions and routinely once weekly, (2) when bacterial superinfection was clinically suspected, or (3) during clinical deterioration or sepsis requiring urgent antibiotic modification, irrespective of cause.

Respiratory samples were analyzed by Gram stain, quantitative bacterial and fungal cultures with susceptibility testing, PCR for Cytomegaly Virus (CMV) and Herpes Simplex Virus (HSV), multiplex PCR for respiratory viruses (at the physician’s discretion), galactomannan testing as well as PCR for Aspergillus and Zygomycetes. In COVID-19 patients, blood was routinely sampled twice weekly for CMV-PCR (EDTA-tubes) and galactomannan testing. Blood cultures were obtained during clinical deterioration, and additional specimens (e.g. catheter tips, urine, wound swabs), were collected as clinically indicated.

In contrast to the meticulous microbiological workup of COVID-19 patients, Influenza patients underwent less frequent testing using the same methods, although only limited to ICU admission and to clinical deterioration. Zygomycetes PCR was rarely performed.

Due to the difficulty of diagnosing bacterial superinfection in ARDS patients, the threshold for additional sampling was intentionally low in the presence of fever, purulent secretions, or clinical deterioration, even without radiologic progression. Clinical deterioration was defined as: (1.) unexplained hemodynamic instability requiring vasopressor support or escalation, (2.) an unexplained increase of minute ventilation and/or worsening gas exchange, or (3.) urgent antibiotic modification due to an intercurrent event. All BAL samples were sent for workup immediately.

### Pulmonary bacterial superinfection

Pulmonary bacterial superinfection was defined by the presence of at least two of the following criteria: leukocytosis (≥ 10/nL) or leukopenia (< 3/nL), purulent tracheal secretions, increased minute ventilation, worsening oxygenation requiring adjustment of ventilator or V-V ECMO, or escalating vasopressor support. A new and persistent pulmonary infiltrate was considered supportive but not mandatory, as identification of a new infiltrate is often challenging in patients with ARDS. Confirmation of pulmonary bacterial superinfection required 2 of the aforementioned clinical criteria and significant bacterial growth in respiratory specimens [15]. Fever was not considered a criterion due to extracorporeal treatment.

Recurrent pulmonary bacterial superinfection, fulfilling VAP criteria (onset after ≥ 48 h after intubation), was defined as recurrence of clinical signs after partial or complete resolution of a previous episode together with significant bacterial growth. The diagnosis was established by the treating physician, reviewed during weekly antimicrobial stewardship rounds, and confirmed by retrospective chart review for the present study. Episodes were classified as relapses if the same pathogen (genus and species) was isolated, or as independent VAP if caused by a different pathogen, consistent with consensus definition [16].

### Viral reactivation and aspergillosis

CMV reactivation (systemic or respiratory) was defined by > 10^3^ copies/mL detected in BAL fluid or blood, following previous publications [13]. Tracheobronchial HSV reactivation was diagnosed with macroscopic bronchoscopic, clinical and microbiological findings. An HSV viral load > 10^4^ copies/mL constituted a sufficient, but not necessary, diagnostic criterion. Pulmonary aspergillosis, including tracheobronchial aspergillosis, was diagnosed using COVID-19 associated pulmonary aspergillosis (CAPA) criteria, applicable to both COVID-19 and influenza patients [17].

### Antimicrobial treatment

Empirical antimicrobial treatment followed current German and international guidelines [18–20]. The threshold for initiating preemptive antifungal therapy was low because early clinical experience during the COVID-19 pandemic suggested frequent invasive aspergillosis and high associated mortality (Table S9). HSV reactivation was treated with acyclovir and CMV reactivation was treated with ganciclovir until PCR negativity. Suspected invasive aspergillosis was generally treated with second- or third-generation azoles. COVID-19 specific therapies followed contemporaneous recommendations, and all influenza patients received oseltamivir for at least 10 days. Glucocorticoids were routinely used in accordance with contemporaneous COVID-19 treatment recommendations. Corticosteroid use was not collected in influenza patients, as their use was generally restricted to severe septic shock because of previously reported associations with increased mortality in influenza-associated ARDS [21].

### Definitions, statistical analysis and laboratory test

Categorical variables are presented as counts and percentages and continuous variables as median (interquartile range). Group comparisons used Chi-square or Fisher’s exact test (n < 5) for categorical variables and the Mann–Whitney U test for continuous variables. Odds ratios were calculated using categorized continuous variables. Because missing data was minimal, no imputation was performed and analyses were based on complete cases. Variables with > 5% missing data were analyzed using complete-case analysis, with the number of evaluable cases reported accordingly. Incidence-density analyses were performed using Poisson regression models.

A p-value < 0.05 was considered statistically significant. To account for multiple comparisons, Bonferroni correction was applied to analyses of the major infectious complications, including pulmonary bacterial superinfection, pulmonary aspergillosis, CMV reactivation, and HSV reactivation. Other subgroup analyses were considered exploratory and are reported descriptive. Analyses were performed using IBM SPSS Statistic software version 25.0 (SPSS Inc. Chicago, IL, USA). Survival was defined as survival to ICU discharge.

## Results

### Baseline characteristics

A total of 147 patients with COVID-19 and 72 patients with Influenza were included. Survival to ICU discharge was 60.5% in the COVID-19 group and 72.2% in the influenza group (p = 0.09). In both cohorts, survivors were younger and had lower Sequential Organ Failure Assessment (SOFA) scores. Among COVID-19 patients, survivors had a shorter interval from symptom onset to ECMO initiation than non-survivors (10 vs. 14 days, p = 0.035). In influenza patients, survivors had shorter times from symptom onset to both intubation and ECMO initiation (Table 1). At ECMO initiation, inflammatory markers were higher in influenza than in COVID-19 patients (Figure S2). No relevant differences were observed in the administration of COVID-19-specific therapies (Table S3). More than 90% of COVID-19 patients received glucocorticoids during their clinical course.

**Table 1:** Baseline characteristics

Variable	COVID-19			p-value	Influenza			p-value	p-value (COVID-19 vs influenza)
	All (n = 147)	Survivor (n = 89)	Non-survivor (n = 58)		All (n = 72)	Survivor (n = 52)	Non-survivor (n = 20)		
Male sex, n (%)	113 (77)	65 (74)	48 (81)	0.252	38 (53)	21 (40)	17 (85)	0.001	< 0.001
Age (years)	55.3 (48.7–61.7)	53.4 (47.7–58.3)	59.1 (53.0–64.3)	< 0.001	55.3 (46–61.3)	52.9 (44.7–59.4)	58.9 (51.4–66.0)	0.022	0.581
Days on V-V ECMO (median (IQR))	23 (14–38)	21 (15–35)	26 (12–40)	0.741	16 (10–23)	15 (10–21)	18 (12–31)	0.250	< 0.001
Body-Mass Index (kg/m^2^)	29.3 (26.2–34.2)	29.3 (26.1–36.9)	29.0 (26.3–32.3)	0.579	28.6 (24.7–34.5)	29.3 (24.7–35.7)	28.1 (24.0–32.4)	0.193	0.537
Sequential Organ Failure Assessment (SOFA)	9 (8–12)	9 (9–10)	11 (8–13)	0.025	13 (10–15)	11 (9–14)	15 (13–17)	< 0.001	< 0.001
Murray score	3.3 (3.3–3.7)	3.3 (3.3–3.7)	3.3 (3.3–3.3)	0.08	3.3 (3.3–3.7)	3.3 (3.3–3.7)	3.3 (3.3–3.7)	0.721	0.154
Pulmonary compliance before V-V ECMO (ml/mbar)	26 (19–33)	25 (18–33)	27 (22–35)	0.202	28 (22–36)	28 (21–35)	30 (23–38)	0.478	0.295
Days from intubation to V-V ECMO	3 (1–8)	2 (1–7)	4 (1–9)	0.231	1 (0–3)	1 (0–4)	1 (1–2)	0.892	0.002
Days from first symptoms to intubation	7 (2–12)	6 (2–11)	8 (3–14)	0.260	1 (0–4)	1 (0–4)	1 (1–5)	0.237	< 0.001
Days from first symptoms to V-V ECMO	12 (7–20)	10 (7–19)	14 (9–20)	0.035	3 (1–9)	4 (1–9)	3 (2–9)	0.834	< 0.001
*Laboratory testing before ECMO*									
Creatinine (mg/dl)	0.9 (0.7–1.5)	0.9 (0.7–1.4)	1.0 (0.7–1.7)	0.734	1.5 (0.8–2.5)	1.3 (0.7–2.0)	2.8 (1.6–3.8)	< 0.001	< 0.001
Blood urea (mg/dl)	57 (37–82)	55 (36–80)	59 (38–86)	0.258	64 (40–101)	50 (37–82)	87 (59–125)	0.011	0.151
Creatine kinase (U/L)	187 (60–446)	209 (57–601)	138 (65–410)	0.356	350 (151–1162)	283 (153–745)	850 (136–2574)	0.130	0.001
LDH (Lactate dehydrogenase) (U/L)	443 (313–648)	442 (312–647)	448 (320–656)	0.845	577 (320–839)	532 (283–702)	705 (539–1418)	0.003	0.088
ASAT (aspartate aminotransferase) (U/L)	56 (41–89)	57 (41–92)	53 (35–89)	0.848	87 (45–1419)	80 (45–129)	130 (41–299)	0.115	0.007
Bilirubin (mg/dl)	0.6 (0.4–1)	0.6 (0.4–0.9)	0.8 (0.5–1.0)	0.007	0.7 (0.4–1.3)	0.6 (0.4–1.1)	1.0 (0.6–2.4)	0.010	0.249
CRP (C-reactive protein) (mg/l)	124 (33–231)	113 (34–209)	151 (31–256)	0.288	182 (98–286)	163 (98–268)	202 (75–290)	0.659	0.008
Procalcitonin (ng/ml)	0.6 (0.24–1.68)	0.6 (0.2–1.1)	0.7 (0.2–2.8)	0.269	2.8 (1.0–11.3)	2.0 (0.9–11.5)	3.5 (2.2–9.0)	0.389	< 0.001
INR (international normalised ratio)	1.1 (1.0–1.2)	1.1 (1–1.2)	1.1 (1.0–1.2)	0.313	1.0 (1.0–1.2)	1.1 (1.0–1.2)	1.0 (0.9–1.3)	0.764	0.183
Fibrinogen (mg/dl)	595 (467–692)	598 (463–699)	588 (483–692)	0.848	466 (381–583)	470 (353–575)	423 (383–608)	0.687	< 0.001
D-Dimers (mg/l)	4.0 (2.0–9.8)	3.0 (2.0–8.5)	4.0 (2.0–11.0)	0.383	5.0 (3.0–9.0)	4.0 (2.0–7.0)	6.5 (4.0–24.3)	0.027	0.345
Platelets (1/nl)	275 (195–377)	290 (213–397)	243 (164–352)	0.021	164 (120–208)	167 (145–228)	141 (85–187)	0.026	0.002
White blood cells (1/nl)	11.7 (8.4–16.4)	11.3 (8–16.5)	12.7 (8.8–16.5)	0.304	11.2 (8.1–16.8)	11.8 (8.3–16.8)	9.3 (7.4–13.8)	0.359	0.674
Neutrophil to lymphocyte ratio*	12.9* (7.8–21.0)	12.9* (7.8–20.5)	12.9* (7.2–22.6)	0.767*	8.62* (4.36 –16.7)	6.3* (3.6–14.6)	11.7* (7.6–31.2)	0.054*	0.006*

Continuous variables are shown as median and IQR 25^th^–75th. Categorized variables are shown as number and percentage of group’s size

*V-V ECMO* veno-venous extracorporeal membrane oxygenation

*Missing data of 19 COVID-19 and 27 Influenza patients

### Pulmonary bacterial superinfections

Pulmonary bacterial superinfections were cumulatively more frequent in COVID-19 than influenza patients, even after adjustment for V-V ECMO duration and pre-ECMO SOFA score (adjusted p = 0.006). Overall, 61% of COVID-19 patients developed a pulmonary bacterial superinfection, compared with 35% of influenza patients (p < 0.001; Table 2, Figure S3). However, incidence-density rates per 1000 ventilation and 1000V-V ECMO days did not differ significantly between groups (Table 3). Rates of initially antibiotic-resistant pathogens were comparable between groups (Table 2).

**Table 2:** Cumulative incidence of pulmonary bacterial superinfections

Variable	COVID-19				Influenza				p-value COVID-19 vs influenza
	All (n = 147)	Survivor (n = 89)	Non-survivor (n = 58)	p-value	All (n = 72)	Survivor (n = 52)	Non-survivor (n = 20)	p-value	
Pulmonary bacterial superinfection, n (% of all)	89 (61)	55 (62)	34 (59)	0.700	25 (35)	20 (39)	5 (25)	0.283	< 0.001*
Initial bacterial pathogen resistant to antibiotics, n (% of all)	28 (31)	13 (15)	15 (26)	0.043	6 (8)	6 (12)	0 (0)	0.160	0.510

Categorized variables are shown as number and percentage of group’s size

*Significance according to adjusted p-value < 0.0125 for major infectious complications

**Table 3:** Incidence-density of pulmonary bacterial superinfections, represented as rate per 1000 days on ventilation and veno-venous extracorporeal membrane oxygenation (V-V ECMO)

	Rate per 1000 days on ventilation COVID-19 vs. influenza				Rate per 1000 days on V-V ECMO COVID-19 vs. influenza			
	COVID -19	Influenza	IRR	p-value	COVID-19	Influenza	IRR	p-value
Pulmonary bacterial superinfections (95% CI)	17.15	15.53	1.10 (0.71–1.72)	0.66	20.27	18.49	1.10 (0.70–1.71)	0.68

*IRR* incidence rate ratio, *CI* confidence interval

**Fig. 1:**
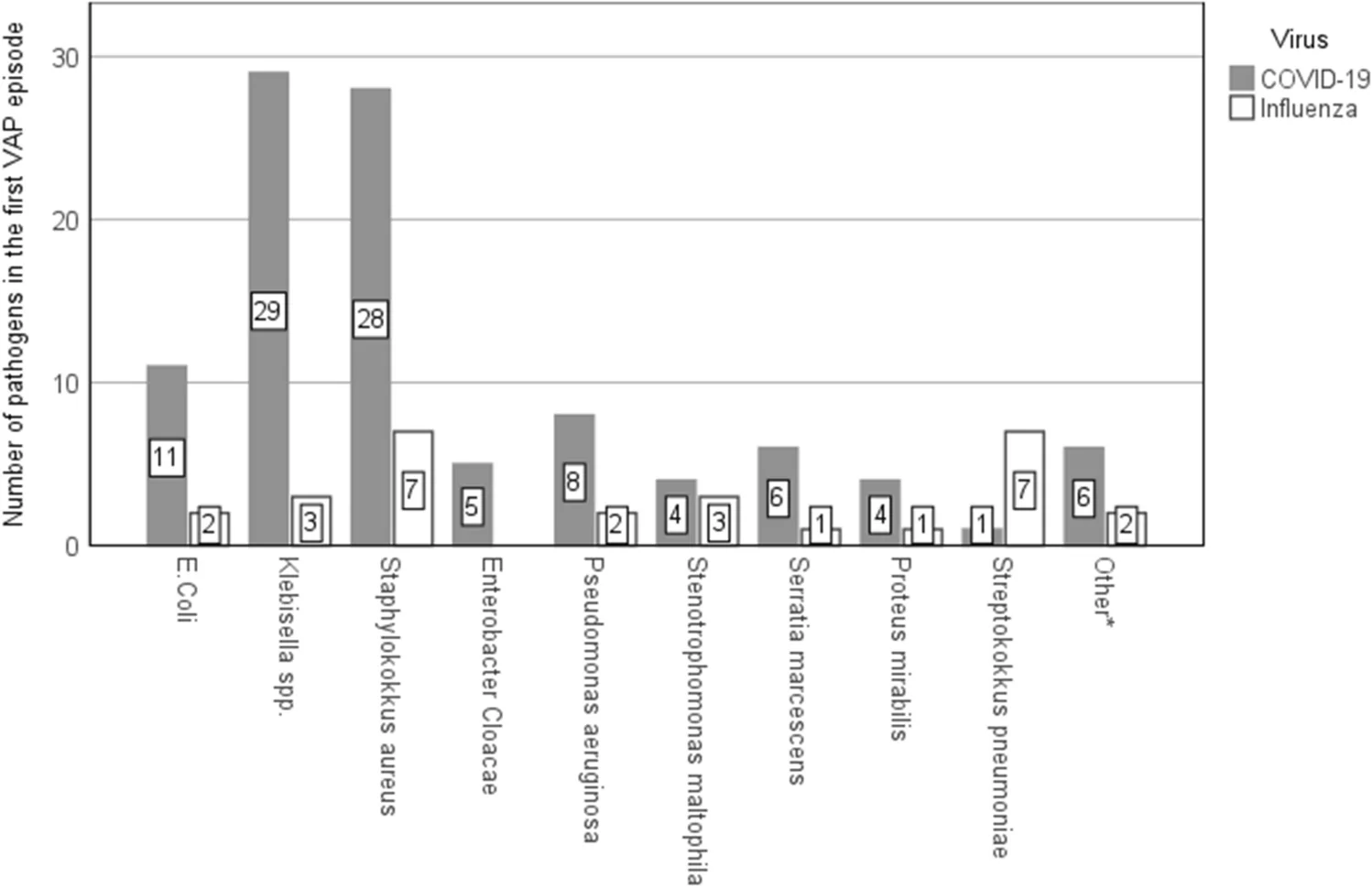
Number of bacterial pathogens in the first episode of pulmonary bacterial superinfection. Values display absolute cases of all detected pathogens (11 COVID-19 patients and 3 Influenza patients with more than one bacterial pathogen in first VAP). *Other: each n = 1: Delftia acidovorans, Moraxella catarrhalis, Moraxella morganii, Raoultella planticola, Serratia rubidaea, Haemophilus influenzae, Legionella spp, Acinetobacter baumanii

During the first episode of pulmonary bacterial superinfection, Klebsiella spp. (28%) and Staphylococcus aureus (27%) predominated in COVID-19 ARDS, whereas Staphylococcus aureus (25%) and Streptococcus pneumoniae (25%) were most common in influenza patients. Klebsiella spp. (11%) were less frequent (Fig. 1, Fig. S4).

The initial bacterial spectrum did not differ between survivors and non-survivors. However, Pseudomonas-associated pulmonary infections occurred significantly more often in COVID-19 non-survivors (p = 0.012) and occurred exclusively as additional VAP episodes. Multiple bacterial pathogens during first pulmonary bacterial superinfection episodes were detected in 11 COVID-19 and 3 influenza patients.

Pulmonary bacterial superinfection occurred earlier after symptom onset in influenza patients (4 vs. 18 days, p = 0.005, Table S5). Antibiotic-resistant isolates were more frequent in COVID-19 non-survivors (p = 0.043) and overall resistance was higher in COVID-19 (Table S1), mainly due to pathogens causing subsequent VAP episodes. Baseline susceptibility profiles during the initial pulmonary bacterial superinfection were similar between groups (Tables 2, 3), with extended-spectrum-β-lactamase (ESBL) production being the most common resistance mechanism.

### Additional pulmonary bacterial infection episodes

Additional bacterial superinfections, exclusively meeting criteria for VAP (onset > 48 h after intubation), occurred only in COVID-19 patients. Twenty-seven patients (18% of the entire cohort; 30% of those with VAP) developed a second pulmonary bacterial infection like VAP, each caused by a different pathogen than the initial episode. Staphylococcus aureus was uncommon in subsequent episodes. Eleven cases were classified as relapses, predominantly due to Klebsiella spp. (64%). Multidrug resistance in Pseudomonas aeruginosa and Klebsiella spp. emerged in 47% of recurrent infections (Fig. S1). In contrast, only one influenza patient developed resistance (ESBL-producing E.coli.).

### Aspergillus superinfection

In the COVID-19 cohort, 26 of 147 patients (18%) had Aspergillus growth in respiratory samples (BALF and bronchial aspirate), without significant impact on survival (p = 0.743, Table 4). In contrast, Aspergillus was detected in 8 influenza patients (11%) and was associated with significantly higher mortality (25% vs 6%, p = 0.020). Probable pulmonary aspergillosis was diagnosed in 48 COVID-19 patients (33%), with 23 deaths (48%), not reaching statistical significance (p = 0.144). Among influenza patients, 16 (22%) met criteria for probable aspergillosis, which was significantly associated with increased mortality (p = 0.024). Overall, 7 died from invasive aspergillosis, including 6 COVID-19 patients (4%) and 1 influenza patient (5%). Aspergillosis occurred earlier in influenza patients (median 11 vs. 23 days after ICU admission, p = 0.001; Table S7). Neither cumulative pulmonary aspergillosis nor incidence-density rates per 1000 ventilation and 1000V-V ECMO days did differ significantly between groups (Table 5). Aspergillus fumigatus was the predominant species, identified in 31 out of 34 positive cultures (91%).

**Table 4:** Aspergillus superinfection in COVID-19 and Influenza patients and definition as probable aspergillosis

	COVID-19				Influenza				p-value COVID-19 vs influenza
	All (n = 147)	Survivor (n = 89)	Non-survivor (n = 58)	p-value	All (n = 72)	Survivor (n = 52)	Non-survivor (n = 20)	p-value	
Positive culture of BALF/aspirate culture, n (%)	26 (18)	15 (17)	11 (19)	0.743	8 (11)	3 (6)	5 (25)	0.020	0.207
Probable aspergillosis, n (%)	48 (33)	25 (28)	23 (40)	0.144	16 (22)	8 (15)	8 (40)	0.024	0.111*
Zygomycetes, n (%)	3 (2)	1 (2)	2 (2)*	0.826	1 (1)	1 (2)	0 (0)	0.343	0.095
Died from aspergillosis, n (%)	6 (4)	0 (0)	6 (10)		1 (1)	0 (0)	1 (5)		

Categorized variables are shown as number and percentage of group’s size

BALF *bronchoalveolar lavage fluid*

*In one patient Zygomycetes was detected in autopsy. *Significance according to adjusted p-value < 0.0125 for major infectious complications

**Table 5:** Incidence-density of COVID-19 Associated Pulmonary Aspergillosis (CAPA) and Influenza Associated Pulmonary Aspergillosis (IAPA), represented as rate per 1000 days on ventilation and veno-venous extracorporeal membrane oxygenation (V-V ECMO)

	Rate per 1000 days on ventilation COVID-19 vs. influenza				Rate per 1000 days on V-V ECMO COVID-19 vs. influenza			
	COVID -19	Influenza	IRR	p-value	COVID-19	Influenza	IRR	p-value
CAPA/IAPA (95% CI)	9.25	9.94	0.93 (0.53–1.64)	0.80	11.14	9.68	0.92 (0.53–1.63)	0.78

*IRR* incidence rate ratio, *CI* confidence interval

### Exploratory observations of preemptive antifungal therapy and ECMO

Antifungal therapy was administered to 127 of 147 COVID-19 patients (86%) and 49 of 72 influenza patients (68%) (Table S9). Overall, Caspofungin was mainly used in COVID-19 patients (110/127, 87%), whereas Voriconazaole was more common in influenza patients (37/49, 76%), reflecting its known effectiveness in invasive aspergillosis. Invasive pulmonary aspergillosis was confirmed before ECMO in 3% of COVID-19 patients (4/147)—classified as CAPA (COVID-19 Associated Pulmonary Aspergillosis)—and 4% of influenza patients (3/72)—classified as IAPA (Influenza Associated Pulmonary Aspergillosis), all of which received targeted antifungal therapy.

**Fig. 2:**
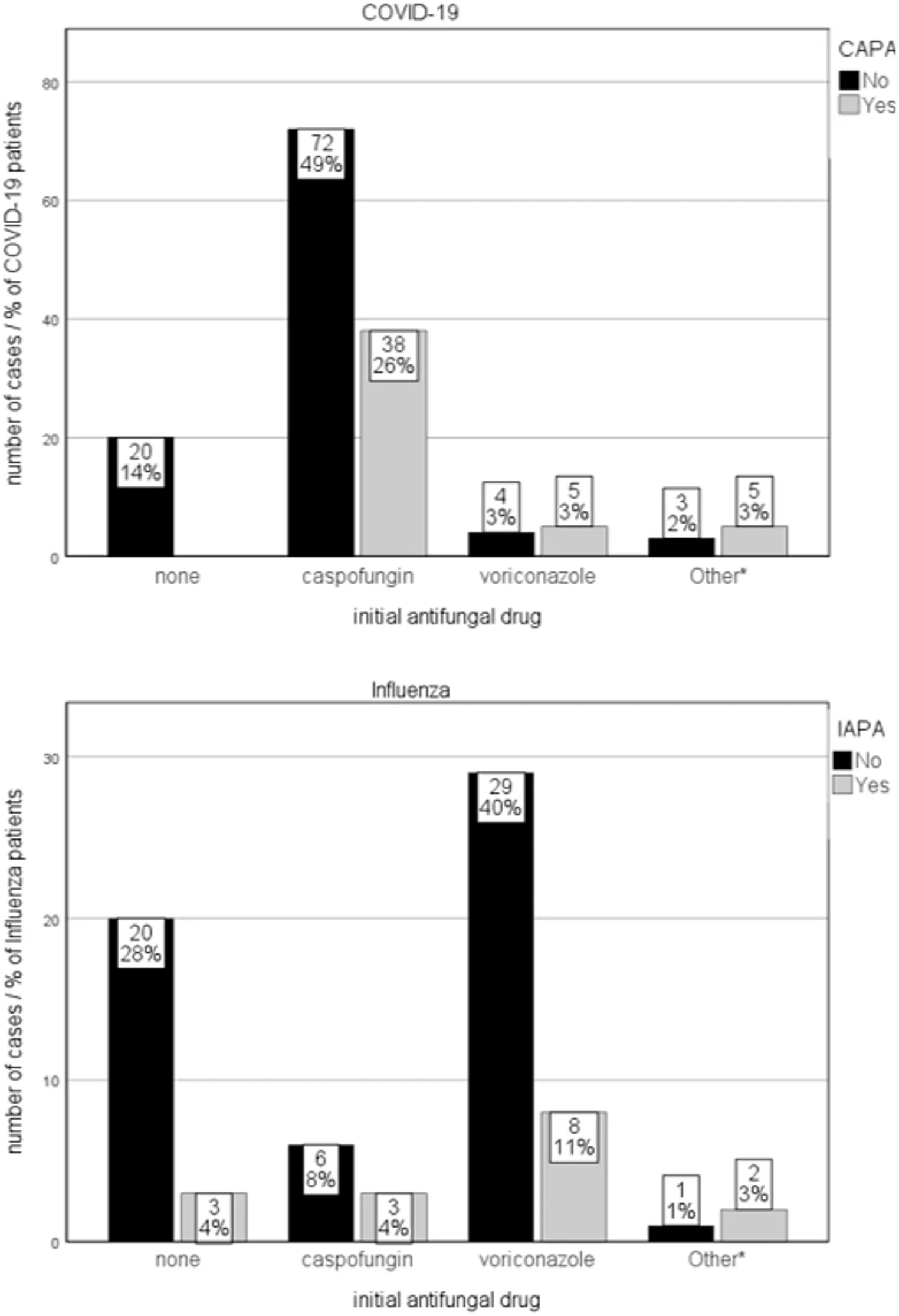
Initial antifungal therapy in COVID-19 and influenza patients meeting diagnostic criteria for pulmonary aspergillosis during ICU stay. *Other: Isavuconazole, Posaconazole, Amphotericin B, Anidulafungin, Caspofungin/Isavuconazole-combination. *CAPA* COVID-19 associated pulmonary aspergillosis, *IAPA* influenza associated pulmonary

Empirical antifungal therapy was initiated in patients with severe respiratory deterioration without microbiological or radiological evidence for aspergillosis in 98 COVID-19 cases (67%) and 25 influenza patients (32%), either before ECMO or within the first five days. Caspofungin was also the main preemptively used agent in COVID-19 patients (85/98, 87%), while Voriconazole was most frequently used in influenza patients (29/72, 40%). (Fig. 2; Table S9).

**Fig. 3:**
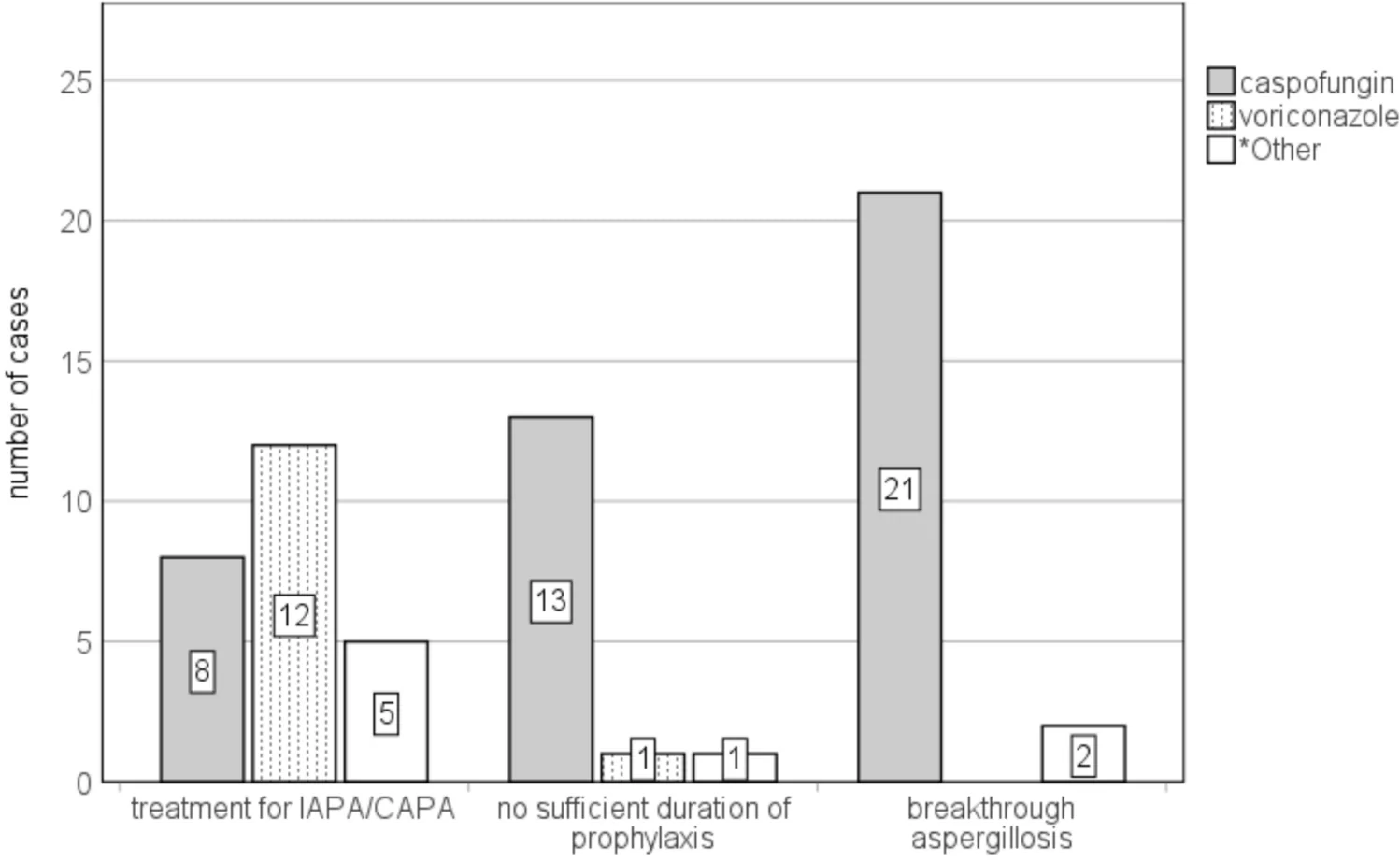
Antifungal therapy in Influenza Associated Pulmonary Aspergillosis (IAPA) and COVID-19 Associated Pulmonary Aspergillosis (CAPA): therapeutic antifungal treatment after diagnosis (left), development of IAPA/CAPA before sufficient duration of preemptive therapy (middle), and breakthrough aspergillosis despite sufficient duration of preemptive antifungal therapy (right). *Other: Isavuconazole, Posaconazole, Amphotericin B

**Fig. 4:**
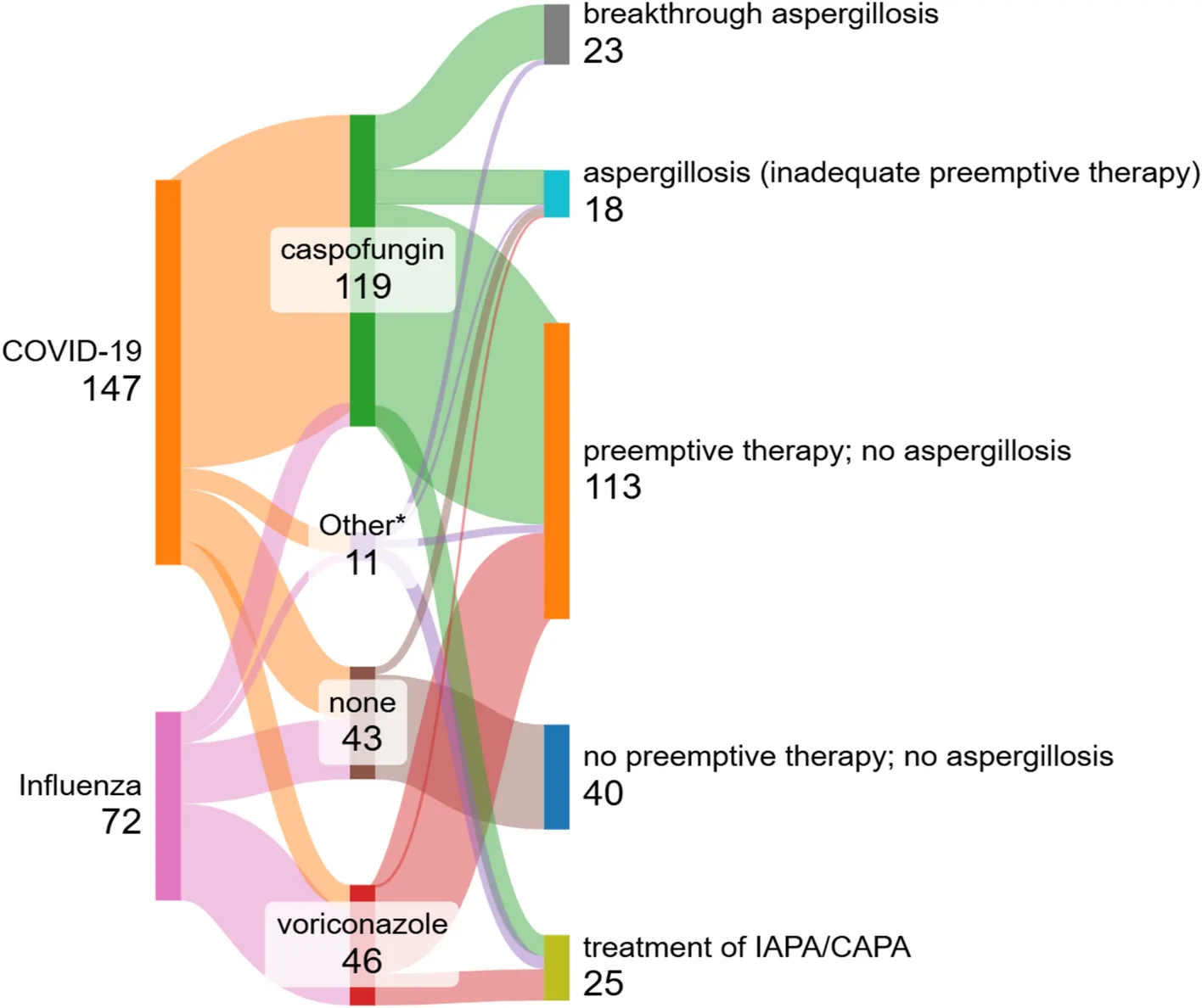
Comparison of antifungal agents in COVID-19 and influenza patients regarding the following outcomes: [1] breakthrough aspergillosis despite adequate duration of preemptive therapy, [2] development of aspergillosis before adequate preemptive therapy duration, [3] adequate preemptive therapy duration without development of aspergillosis, [4] no preemptive therapy without developing aspergillosis, and [5] primary treatment of Influenza Associated Pulmonary Aspergillosis (IAPA) or COVID-19 Associated Pulmonary Aspergillosis (CAPA). No cases of breakthrough aspergillosis were observed in patients receiving Voriconazole. *Other: Isavuconazole, Posaconazole, Amphotericin B, Anidulafungin, Caspofungin/Isavuconazole-combination

Breakthrough invasive aspergillosis (≥ 7 days of continuous antifungal therapy) occurred in 22 COVID-19 patients (15%) and 1 influenza patient (1%). Almost all breakthrough cases in COVID-19 occurred during Caspofungin (21/22, 95%), whereas none occurred with Voriconazole (0/33). Preemptive therapy without progression for proven aspergillosis was observed in 79 COVID-19 patients (54%) and 36 influenza patients (54%) (Fig. 4). Stratified by agent, breakthrough aspergillosis occurred in 22% (21/93) of Caspofungin-treated patients and in none receiving Voriconazole (0/33) (Fig. 3; Table S9). Exploratory incidence-density rates of breakthrough aspergillosis per 1000 ventilation and 1000V-V ECMO days did not differ significantly between groups, but a numerical difference was observed (Table 6) (Fig. 4).

**Table 6:** Incidence-density breakthrough aspergillosis, represented as rate per 1000 days on ventilation and veno-venous extracorporeal membrane Oxygenation (V-V ECMO)

	Rate per 1000 days on ventilation COVID-19 vs. influenza				Rate per 1000 days on V-V ECMO COVID-19 vs. influenza			
	COVID -19	Influenza	IRR	p-value	COVID-19	Influenza	IRR	p-value
Breakthrough aspergillosis	4.24	0.62	6.84 (0.93–50.40)	0.059	5.11	0.74	6.91 (0.94–50.90)	0.058

*IRR* incidence rate ratio, *CI* confidence interval

Twenty COVID-19 patients (13%) and 23 influenza patients (31%) did not receive antifungal therapy and did not meet the criteria for probable CAPA or IAPA. Among these, seven COVID-19 patients (35%) died without antifungal treatment, though none developed probable CAPA. In contrast, 3 influenza patients met criteria for probable IAPA without treatment of whom 2 (67%) survived. In COVID-19 patients, aspergillosis was associated with male sex, older age, longer ECMO duration and higher pre-ECMO C-reactive protein (CRP) levels (Table S3). Survivors with CAPA were younger, initiated ECMO earlier, and had lower alkaline phosphatase levels before cannulation. No significant baseline differences were observed in influenza patients.

### Viral reactivations

Systemic CMV reactivation occurred in 26 COVID-19 patients (18%) without significant impact on survival (16% vs. 14%, p = 0.441) and in 2 influenza patients (3%) (p = 0.596) (Table 7). Pulmonary CMV and HSV reactivation were not associated with survival in either cohort. However, pulmonary CMV (p = 0.009) and HSV (p = 0.005) reactivation were more frequent in COVID-19 patients.

**Table 7:** Cumulative viral reactivations in COVID-19 and influenza patients with systemic and pulmonary cytomegalovirus (CMV) reactivation and pulmonary herpes simplex (HSV) reactivation

	COVID-19				Influenza				p-value COVID-19 vs influenza
	All (n = 147)	Survivor (n = 89)	Non-survivor (n = 58)	p-value	All (n = 72)	Survivor (n = 52)	Non-survivor (n = 20)	p-value	
Systemic CMV reactivation, n (%)	26 (18)	14 (16)	12 (21)	0.441	2 (3)	1 (2)	1 (5)	0.596	0.002*
Pulmonary CMV reactivation, n (%)	28 (19)	16 (18)	12 (21)	0.682	3 (4)	2 (4)	1 (5)	0.835	0.009*
Pulmonary HSV reactivation, n (%)	72 (49)	43 (48)	29 (50)	0.841	19 (26)	14 (27)	5 (25)	0.949	0.005*

Categorized variables are presented as number and percentage of the respective group

*Significance according to adjusted p-value < 0.0125 for major infectious complications

**Fig. 5:**
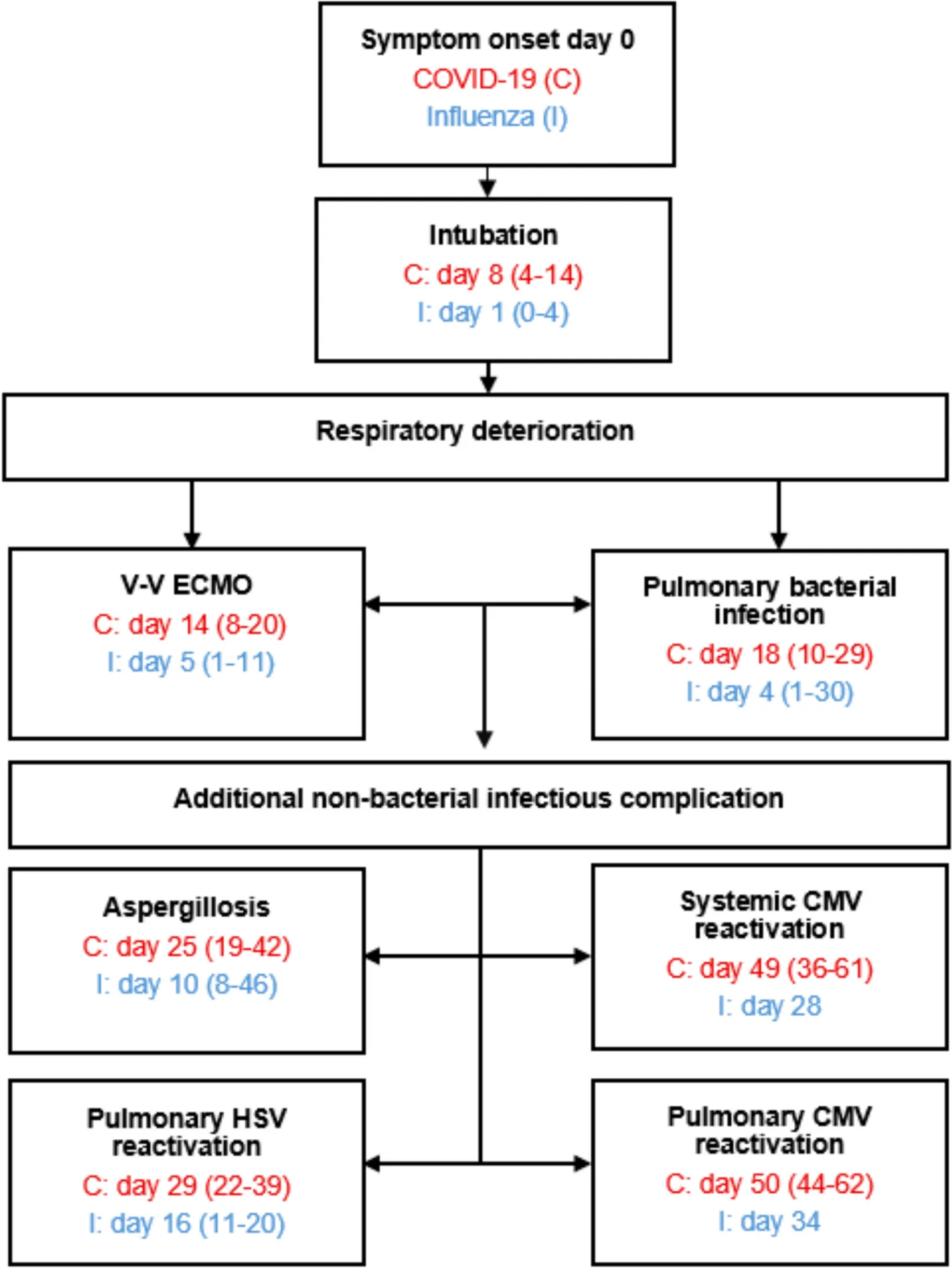
Clinical course COVID-19 (“C”, red) and influenza (“I”, blue) patients requiring veno-venous extracorporeal membrane oxygenation (V-V ECMO) and developing additional infectious complications. Reference time is onset of first symptoms. Time intervals are presented as median and Interquartile range (IQR 25^th^–75th percentile). *CMV* cytomegalovirus, *HSV* herpes simplex virus

Tracheobronchial HSV reactivation occurred in 49% of COVID-19 patients and was cumulative significantly more frequent than in influenza patients (26%; adjusted for V-V ECMO duration and SOFA Score, p = 0.031). HSV tracheobronchitis developed earlier in influenza than COVID-19 patients (16 vs. 27 days, p = 0.001). However, incidence-density rates of systemic and pulmonary CMV reactivation as well as pulmonary HSV reactivation per 1000 ventilation and 1000V-V ECMO days did not differ significantly between groups (Table 8). Infection timelines and survival are shown in Fig. 5 and Fig. S1A, B.

**Table 8:** Incidence-density of systemic and pulmonary cytomegalovirus (CMV) reactivation and pulmonary herpes simplex (HSV) reactivation, represented as rate per 1000 days on ventilation and 1000 days on veno-venous extracorporeal membrane oxygenation (V-V ECMO)

	Rate per 1000 days on ventilation COVID-19 vs. Influenza				Rate per 1000 days on V-V ECMO COVID-19 vs. Influenza			
	COVID -19	Influenza	IRR	p-value	COVID-19	Influenza	IRR	p-value
Systemic CMV reactivation	5.01	1.24	1.36 (0.33–5.79)	0.66	6.03	1.48	1.24 (0.3–5.24)	0.77
Pulmonary CMV reactivation	5.40	1.86	2.52 (0.77–8.27)	0.13	6.50	2.22	2.48 (0.75–8.16)	0.14
Pulmonary HSV reactivation	13.87	11.80	1.12 (0.68–1.86)	0.66	16.71	14.05	1.11 (0.67–1.85)	0.68

*IRR* incidence rate ratio, *CI* confidence interval

### Clinical complications and mortality

The impact of clinical complications on mortality in COVID-19 and influenza patients was assessed using univariable and multivariable analysis adjusted for age, SOFA score, BMI and sex (Table 9). Among complications, only renal replacement therapy (RRT) and intracranial hemorrhage (ICH) were independently associated with mortality. Age, BMI and sex were also significant predictors. Underlying viral infections was a strong determinant, with influenza being associated with a markedly lower risk for death on ICU than COVID-19 after adjustment (adjusted OR 0.16, CI 0.04–0.57, p = 0.005).

**Table 9:** Univariable and multivariable analyses of factors associated with ICU mortality

Factor	Odds ratio (OR) univariable (death on ICU) (95% CI)	p-value	Adjusted odds ratio (AOR) multivariable (death on ICU) (95% CI)	p-value
Virus (COVID-19 vs influenza)	0.59 (0.32–1.09)	0.092	0.16 (0.04 –0.57)	0.005
Probable aspergillosis	2.16 (1.19–3.92)	0.012	1.51 (0.64–3.57)	0.351
Pulmonary bacterial superinfection	0.88 (0.51–1.53)	0.651	0,74 (0.33–1.67)	0.474
Positive blood culture	1.86 (1.06 –3.26)	0.031	1.66 (0.68–4.01)	0.264
HSV tracheobronchitis	1.15 (0.65 –2.02)	0.627	0.52 (0.23–1.21)	0.13
CMV reactivation	1.40 (0.68–2.85)	0.359	0.92 (0.36–2.36)	0.872
RRT	4.52 (2.50–8.19)	< 0.001	5.02 (1.90–13.32)	0.001
Pulmonary embolism	0.942 (0.50 –1.78)	0.855	1.05 (0.43–2.59)	0.92
ICH	5.13 (2.10–12.51)	< 0.001	5.60 (1.81–17.38)	0.003
Stroke	2.66 (0.72–9.79)	0.141	1.77 (0.35–8.90)	0.488
Age	1.06 (1.03–1.10)	< 0.001	1.05 (1.00–1.10)	0.047
SOFA pre ECMO	1.15 (1.05–1.26)	0.002	1.16 (1.00–1.34)	0.059
BMI	0.97 (0.93–1.01)	0.108	0.91 (0.34–0.98)	0.014
Male sex	0.35 (0.18–0.69)	0.002	0.34 (0.13–0.88)	0.026

Renal replacement therapy (RRT), intracranial hemorrhage (ICH), age, BMI (body mass index), male sex and COVID-19 (vs. Influenza) were independently associated with mortality

*HSV* herpes simplex, *CMV* cytomegalovirus, *SOFA* sequential organ failure assessment, *V-V ECMO veno-venous e*xtracorporeal membrane oxygenation

## Discussion

Microbiological data on patients receiving V-V ECMO for viral ARDS remain limited. In this retrospective single-center study of a comparatively large cohort, we observed high rates of pulmonary bacterial superinfection in both COVID-19 (61%) and influenza (35%) patients. However, after adjustment for ventilation and V-V ECMO days, incidence-density rates were not significantly different between groups. Nevertheless, notable differences were observed in pathogen distribution, timing of additional non-bacterial infectious complications, and a hypothesis-generating differential response to preemptive antifungal therapy.

The first pulmonary bacterial infection episode occurred earlier in influenza than in COVID-19 patients, consistent with consensus definitions for VAP [16]. Secondary, non-bacterial infections, including aspergillosis, CMV, and HSV reactivation, also occurred earlier in influenza patients. Notably, breakthrough aspergillosis was less frequent in patients receiving Voriconazole than caspofungin, with 96% (21/22) of breakthrough cases occurring during caspofungin therapy. However, this observation must be interpreted very cautiously, as Voriconazole was predominantly used in influenza patients, whereas Caspofungin predominated in COVID-19. Furthermore, microbiological workup was more extensive in COVID-19 than in influenza patients, and the study was not designed to compare antifungal treatment strategies directly.

Reported VAP rates in COVID-19 patients reach up to 86% [6–9], exceeding those in ARDS without ECMO (20%–36%) [22, 23], also compared to previously described COVID-19 patients requiring V-V ECMO [24]. In our cohort, the first bacterial pulmonary infection, most often but not exclusively fulfilling VAP criteria, occurred after a median of 8 days (IQR 2–19) of invasive ventilation, consistent with previous reports [9, 22, 23]. The first pulmonary bacterial superinfection in COVID-19 was mainly caused by Klebsiella spp. (28%), Staphylococcus aureus (27%) and E. coli (11%), differing from published data showing higher Pseudomonas aeruginosa (16%) [25] and lower Staphylococcus aureus [9] and Klebsiella spp. rates [10]. Pathogens causing subsequent pulmonary bacterial superinfections, all of which fulfilled VAP criteria, also differed, with the predominance of Klebsiella spp., in contrast to previously reported dominance of Pseudomonas [9].

Notably, influenza patients did not develop recurrent pulmonary bacterial superinfection, and pulmonary bacterial superinfection in both cohorts occurred close to ECMO initiation, suggesting a potential role in clinical deterioration leading to ECMO support. This highlights the need for early and comprehensive microbiological diagnostics, even in the absence of overt clinical worsening.

The low prevalence of multidrug-resistant pathogens during the initial pulmonary bacterial superinfection episode in COVID-19 patients (3%) contrasts with European data [26] and may reflect local epidemiology. ESBL development occurred in 31% of initial pathogens, consistent with prior reports [9]. Differences in bacterial spectra between COVID-19 and influenza patients with a higher rate of gram-positive pathogens, may be related to earlier onset of pulmonary bacterial superinfection in influenza [27, 28]. Initial pathogen profiles aligned with current guidelines for antibiotic therapy of VAP [19, 29], whereas resistance emerging later was predominantly MDR. The high frequency of Klebsiella spp. in recurrent and relapsing VAP supports consideration of prolonged therapy and microbiological reassessment before antibiotic discontinuation, balanced against the risk of prolonged treatment.

Diagnosing invasive aspergillosis in ICU patients remains challenging, as traditional criteria developed for immunocompromised hosts may not apply [30]. Updated EORTC criteria reflect growing recognition of IAPA and CAPA [31]. Aspergillus fumigatus was the predominant species. Pathogenesis is multifactorial, involving impaired mucociliary clearance due to mechanical ventilation, inflammation, corticosteroid therapy, and host factors [32]. Aspergillosis occurred in 33% of COVID-19 and 22% of influenza patients, consistent with previously reported rates Mortality among COVID-19 patients with aspergillosis did not differ between survivors and non-survivors, and only 25% died directly because of aspergillosis [34, 35, 36]. As the incidence of probable CAPA/IAPA is likely underestimated [37], the observed mortality approaching 50% should not be interpreted as treatment futility, and aspergillosis alone should not justify treatment limitation [38, 39].

Given overlapping risk factors and widespread corticosteroid use, preemptive antifungal may warrant further investigation in selected high-risk patients [40]. In this hypothesis-generating observational analysis, breakthrough aspergillosis occurred more frequently during caspofungin (22% breakthrough rate) than voriconazole therapy. However, this finding should be interpreted with substantial caution, as antifungal selection was not randomized, differed between COVID-19 and influenza patients, and the study was not designed to compare antifungal strategies. Therefore, further data on antifungal therapy, especially with regards to potential pharmacokinetic alterations during V-V ECMO were not systematically collected and require further investigation. Current guidelines continue to recommend Voriconazole or Isavuconazole as first-line therapy for invasive aspergillosis in high-risk immunocompromised patients. Therapeutic drug monitoring is advised due to CYP-mediated interactions.

Earlier onset in our cohort of IAPA compared to CAPA (11 days vs. 23) has been previously described [37]. Diagnoses in our study occurred later than in prior reports, likely reflecting different reference timing being symptom-based rather than ICU-based. Mucormycosis was rare in our patients (2%) as well as reported (1%) [33], suggesting routine prophylaxis does not need to cover it, although regular screening remains important.

CMV reactivation rates (18–19%) were comparable to prior reports both in non-COVID-19 populations and COVID-19 patients requiring V-V ECMO [13, 14]. HSV reactivation (49%) exceeded published rates in COVID-19 patients with ECMO (14% (6)) and without ECMO support (26% (12)), potentially reflecting exclusive respiratory sampling. Viral reactivation occurred later in COVID-19 than in influenza patients, suggesting distinct immune responses, supported by higher inflammatory markers in influenza despite substantially higher adjusted odds of ICU mortality in COVID-19. The mechanisms underlying these differences remain unclear. Importantly, superinfection was not independently associated with mortality in multivariable analysis, possibly reflecting the comparatively large cohort and higher number of aspergillosis survivors compared with previous studies [6, 8].

### Strengths and limitations

The main limitations of this study are its retrospective, single-center design and the limited sample size. Nevertheless, it represents one of the largest analyses comparing microbiological complications in patients receiving V-V ECMO for COVID-19 and influenza. Importantly, microbiological workups differed substantially between groups, with COVID-19 patients undergoing more systematic and comprehensive diagnostics, whereas testing in influenza patients was less frequent and predominantly initiated in response to clinical deterioration. Consequently, infectious complications may have been underestimated in influenza patients. Furthermore, COVID-19 patients had substantially longer durations of mechanical ventilation and V-V ECMO support, which may have increased the opportunity to detect later infectious complications. Although incidence-density analyses were performed to account for differences in exposure time, residual survival ascertainment bias cannot be excluded. In addition, analyses regarding preemptive antifungal therapy were retrospective and exploratory and lacked sufficient power for definitive conclusions.

## Conclusion

Superinfections were common in patients receiving V-V ECMO for viral ARDS and showed distinct microbiological patterns in COVID-19 and influenza. Interpretation of infection frequencies requires caution, as microbiological surveillance was more systematic in COVID-19 patients, whereas testing in influenza patients was less frequent and primarily triggered by clinical deterioration, potentially leading to underascertainment. Initial pulmonary bacterial superinfections frequently occurred around the time of ECMO initiation. Predominant pathogens were Klebsiella spp. (28%), Staphylococcus aureus (27%) and E. coli (11%) in COVID-19 patients, and Staphylococcus aureus (25%) and Streptococcus pneumoniae (25%) in influenza patients.

Recurrent pulmonary bacterial superinfections, all fulfilling VAP criteria, occurred exclusively in COVID-19 patients and were mainly associated with Klebsiella spp. Given the high incidence of fungal infections in viral ARDS, the role of preemptive antifungal therapy remains uncertain and warrants further investigation.

## Supplementary Information

Below is the link to the electronic supplementary material.

Supplementary file1 (DOCX 428 KB)

## Data Availability

All data supporting the findings of this study are available within the paper and its Supplementary Information. Full raw data is available upon request.
