# Supplementary material for: Pathogen patterns in viral ARDS on V-V ECMO: a single-center experience
Source: Infection. 2026 Jul 15;54(5):2549–62. doi: 10.1007/s15010-026-02879-8 (PMC13727879; doi:10.1007/s15010-026-02879-8)
Supplement: Supplementary file 1 [file 15010_2026_2879_MOESM1_ESM.docx]

**Supplementary:**

List of figures:

[Figure 1: Number of bacterial pathogens in the first episode of pulmonary bacterial superinfection. Values display absolute cases of all detected pathogens	13](#_Toc232007610)

[Figure 2: Initial antifungal therapy in COVID-19 and influenza patients meeting diagnostic criteria for pulmonary aspergillosis during ICU stay.	17](#_Toc232007611)

[Figure 3: Antifungal therapy in Influenza Associated Pulmonary Aspergillosis (IAPA) and COVID-19 Associated Pulmonary Aspergillosis (CAPA):.	18](#_Toc232007612)

[Figure 4: Comparison of antifungal agents in COVID-19 and influenza patients regarding the following outcomes: [1.] breakthrough aspergillosis despite adequate duration of preemptive therapy, [2.] development of aspergillosis before adequate preemptive therapy duration, [3.] adequate preemptive therapy duration without development of aspergillosis, [4.] no preemptive therapy without developing aspergillosis, and [5.] primary treatment of Influenza Associated Pulmonary Aspergillosis (IAPA) or COVID-19 Associated Pulmonary Aspergillosis (CAPA)..	20](#_Toc232007613)

[Figure 5: Clinical course COVID-19 (“C”, red) and influenza (“I”, blue) patients requiring veno-venous extracorporeal membrane oxygenation (V-V ECMO) and developing additional infectious complications.	23](#_Toc232007614)

List of tables:

Table 1: Baseline characteristics.………………………………………………………………………………………..…10

Table 2A: Cumulative incidence of pulmonary bacterial superinfections ………………..…………………………….12

Table 2B:: Incidence-density of pulmonary bacterial superinfections, represented as rate per 1000 days on ventilation and veno-venous extracorporeal membrane oxygenation (V-V ECMO)………………………………..…12

Table 3A: Aspergillus superinfection in COVID-19 and Influenza patients and definition as probable Aspergillosis. ………………………………………………………………………………………………………..………………………..15

Table 3B: Incidence-density of COVID-19 Associated Pulmonary Aspergillosis (CAPA) and Influenza Associated Pulmonary Aspergillosis (IAPA), represented as rate per 1000 days on ventilation and veno-venous extracorporeal membrane oxygenation (V-V ECMO)…………………………………………. ………………………..15

Table 4: Incidence-density breakthrough aspergillosis, represented as rate per 1000 days on ventilation and veno-venous extracorporeal membrane Oxygenation (V-V ECMO)……………………………………………………….…19
Table 5A: Cumulative viral reactivations in COVID-19 and Influenza patients with systemic and pulmonary cytomegalovirus (CMV) reactivation and pulmonary herpes simplex (HSV) reactivation…………………………... 21
Table 5B: Table 5B: Incidence-density of systemic and pulmonary cytomegalovirus (CMV) reactivation and pulmonary herpes simplex (HSV) reactivation, represented as rate per 1000 days on ventilation and 1000 days on veno-venous extracorporeal membrane oxygenation (V-V ECMO)……………………………………………………21

Table 6: Univariable and multivariable analyses of factors associated with ICU mortality. ….……………………..24

**Supplementary Chapter: Bloodstream infections**

Bloodstream infections

Methods

Growth of coagulase negative staphylococci (CNS) in blood cultures was classified as probable or possible clinical significance, or as contamination, according to published criteria. In cases of repeated CNS isolation, infective endocarditis was excluded by transesophageal echocardiography. Growth of bacterial species other than CNS was considered clinically significant.

The primary focus of bloodstream pathogens was determined by comparison with microbiological findings from specimens collected at the same time (e.g. BALF, urine, bile, catheter tips). If the same pathogen was isolated from blood and another site, that site was considered as the primary infection focus. When biliary fluid was unavailable, biliary duct infection was diagnosed based on typical laboratory findings (elevated bilirubin, biliary enzymes (GGT, ALP) at the time of bacteriemia and subsequent diagnosis of secondary sclerosing cholangitis (SSC).

Results

Overall, 78 COVID-19 patients (52%) had at least one positive blood culture, with no difference between survivors and non-survivors. In comparison, 13 influenza patients (18%) had positive blood cultures, also without survival differences. The rate of positive blood cultures was significantly higher in COVID-19 than in influenza patients (p=0.001). Among COVID-19 patients, CNS was the most frequently detected bloodstream pathogen, followed by *Enterococcus spp.* (n=21) (Table S6).

In the COVID-19 group, 72 blood cultures were CNS-positive: 34 (47%) were classified as contamination, 22 (31%) as possible infection and 16 (22%) as probable infection. Among influenza patients, 14 CNS-positive blood cultures were identified, with 3 (21%) classified as contamination, 7 (50%) as possible infection and 4 (29%) as probable infection. Blood cultures were assessed individually, including sequential cultures from the same patients.

The most common primary sites of infection were the biliary tract (n=12) and respiratory system (n=10). Among patients with Enterococci bacteremia, the biliary tract was the source in 10 cases (48%) (Table S2). Enterococcus spp. detection was significantly associated with SSC (p<0.001). In contrast, no clear infection source was identified in influenza patients. Candidemia occurred in five COVID-19 patients, exclusively in non-survivors (p=0.013).

Discussion

In this cohort, 33% of COVID-19 patients had clinically significant positive blood cultures, which is lower than reported elsewhere (8). Among influenza patients, 13% had clinically significant bloodstream infections. Pulmonary bacterial infection accounted for 20% of bloodstream infections, highlighting its clinical relevance. The high frequency of Enterococcus spp., with nearly half of it (48%) originating from the biliary tract, underscores the importance of SSC. The candidemia rate (6%) and associated mortality (100%) in COVID-19 patients were consistent with previous reports (33).

|  | COVID-19 | | | | Influenza | | | |  |
| --- | --- | --- | --- | --- | --- | --- | --- | --- | --- |
|  | All with VAP (n=89) | Survivor (n=55) | Non-Sur-vivor (n=34) | p-value | All (n=25) | Survivor  (n=20) | Non-Survivor  (n= 5) | p-value | p-value  (COVID-19 vs Influenza) |
| Staphylococcus aureus | 29 (33) | 20 (36) | 9 (26) | 0.333 | 7 (28) | 7 (35) | 0 (0) | 0.119 | 0.663 |
| MRSA | 1 (1) | 1 (2) | 0 (0) | 0.429 | 2 (8) | 2 (10) | 0 (0) | 0.461 | 0.058 |
| Enterobacter spp. | 8 (7) | 5 (9) | 3 (9) | 0.585 | 0 (0) | 0 (0) | 0 (0) | - | 0.148 |
| Klebsiella spp.* | 40 (45) | 22 (40) | 18 (53) | 0.356 | 3 (12) | 3 (15) | 0 (0) | 0.356 | 0.004 |
| Klebsiella aerogenes | 11 (12) | 5 (9) | 6 (18) | 0.233 | 1 (4) | 1 (4) | 0 (0) | 0.610 | 0.229 |
| 4 MRGN Klebsiella aerogenes | 2 (2) | 1 (2) | 1 (3) | 0.728 | 0 (0) | 0 (0) | 0 (0) | - | 0.450 |
| Klebsiella oxytoca | 7 (8) | 5 (9) | 2 (6) | 0.585 | 2 (8) | 2 (10) | 0 (0) | 0.461 | 0.982 |
| Klebsiella pneumoniae | 22 (25) | 12 (22) | 10 (30) | 0.615 | 0 (0) | 0 (0) | 0 (0) | - | 0.007 |
| 3 MRGN Klebsiella pneumoniae | 1 (1) | 1 (2) | 0 (0) | 0.411 | - | - | - | - | 0.594 |
| Klebsiella ozaneae | 1 (1) | 1 (2) | 0 (0) | 0.429 | 0 (0) | 0 (0) | 0 (0) | - | 0.594 |
| Pseudomonas aeruginosa | 11 (12) | 3 (5) | 8 (24) | 0.012 | 2 (8) | 1 (5) | 1 (20) | 0.269 | 0.545 |
| 3 MRGN Pseudomonas aeruginosa | 4 (5) | 1 (2) | 3 (9) | 0.121 | 1 (4) | 1(5) | 0 (0) | 0.610 | 0.915 |
| 4 MRGN Pseudomonas aeruginosa | 3 (3) | 1 (2) | 2 (6) | 0.302 | 0 (0) | 0 (0) | 0 (0) | - | 0.352 |
| E. coli | 13 (15) | 9 (16) | 4 (12) | 0.551 | 2 (8) | 2 (10) | 0 (0) | 0.461 | 0.388 |
| Haemophilus influenzae | -- | - | - | - | 1 (4) | 1 (5) | 0 (0) | 0.610 | 0.058 |
| Proteus mirabilis | 5 (6) | 1 (2) | 4 (12) | 0.048 | 1 (4) | 1 (5) | 0 (0) | 0.610 | 0.749 |
| Moraxella catarrhalis | 1 (1) | 1 (2) | 0 (0) | 0.429 | - | - | - | - | 0.594 |
| Morganella morganii | 1 (1) | 0 (0) | 1 (3) | 0.201 | - | - | - | - | 0.594 |
| Stenotrophomonas maltophila | 5 (6) | 2 (4) | 3 (9) | 0.619 | 3 (12) | 1 (5) | 2 (40) | 0.031 | 0.167 |
| Serratia marcescens | 6 (7) | 3 (5) | 3 (9) | 0.538 | 1 (4) | 1 (5) | 0 (0) | 0.610 | 0.614 |
| Streptococcus pneumoniae | 1 (1) | 1 (2) | 0 (0) | 0.429 | 7 (28) | 6 (30) | 1 (20) | 0.656 | <0.001 |
| Serratia rubideae | 1 (1) | 1 (2) | 0 (0) | 0.429 | 0 (0) | 0 (0) | 0 (0) | - | 0.594 |
| Delftia acidovorans | 1 (1) | 1 (2) | 0 (0) | 0.429 | 0 (0) | 0 (0) | 0 (0) | - | 0.594 |
| Raoultella planticola | 1 (1) | 1 (2) | 0 (0) | 0.429 | 0 (0) | 0 (0) | 0 (0) | - | 0.594 |
| Acinetobacter baumanii | 1 (1) | 0 (0) | 1 (3) | 0.201 | 0 (0) | 0 (0) | 0 (0) | - | 0.594 |

Table S 1: Bacterial species detected in respiratory material during veno-venous extracorporeal membrane oxygenation (V-V ECMO). All bacterial pathogens detected in respiratory material in patients treated with V-V ECMO due to COVID-19 adult respiratory distress syndrome (ARDS) shown as number and percentage of group’s size.
Abbreviations: MRSA = Methicillin-resistant Staphylococcus aureus, MRGN = multi-resistant Gram-negative

| Bacterial pathogen in blood culture | Focus |
| --- | --- |
| Escherichia Coli (n=1) | Urinary tract |
| Acinetobacter baumanii (n=1) | Respiratory tract |
| Serratia marcescens (n=1) | Respiratory tract |
| Proteus mirabilis (n=1) | Respiratory tract |
| Enterobacter cloacae (n=1) | Respiratory tract |
| Klebsiella pneumoniae (n=2) | Respiratory tract, Urinary tract |
| Pseudomonas aeruginosa (n=2) | Respiratory tract, unknown |
| Staphylococcus aureus (n=5) | Respiratory tract (n=4), unknown |
| Enterococcus spp. (n=21) | Biliary tract (n=10), urinary tract (n=3), infected vascular prothesis (n=1), unknown (n=7) |
| Candida spp. (n=5) | Biliary tract (n=2), unknown (n=3) |

Table S 2: Bacterial pathogens in blood cultures and suspected focus in COVID-19 patients excluding Coagulase-negative staphylococci (CNS).

.

| Aspergillosis | | | | | | | | | |
| --- | --- | --- | --- | --- | --- | --- | --- | --- | --- |
|  | COVID-19 | | | | Influenza | | | |  |
|  | All (n=147) | Aspergillosis (n=48) | No Aspergillosis (n=99) | p-value | All  (n=72) | Aspergillosis  (n=16) | No Aspergillosis  (n=56) | p-value | p-value  (COVID-19 vs Influenza) |
| Survivor,  n (%) | 89 (61) | 25 (52) | 64 (65) | 0.144 | 52 (72) | 8 (50) | 44 (79) | 0.024 | 0.885 |
| Male sex,  n (%) | 113 (77) | 43 (90) | 70 (70) | 0.011 | 38 (53) | 10 (63) | 28 (50) | 0.377 | 0.013 |
| Age (years) | 55.3 (48.7 – 61.7) | 60.7 (53.8 – 64.5) | 53.7 (48.1 – 58.1) | <0.001 | 55.3 (46 – 61.3) | 56.4 (47.7 – 64.5) | 54.3 (45.1 – 60.9) | 0.650 | 0.303 |
| Days on V-V ECMO | 23 (14 – 38) | 28 (19 – 61) | 21 (11 – 35) | 0.005 | 16 (10 – 23) | 16 (6 – 25) | 16 (11 – 22) | 0.755 | 0.003 |
| Size (cm) | 175 (170 – 180) | 175 (170 – 180) | 172 (170 – 180) | 0.038 | 170 (165 – 180) | 170 (168 – 180) | 170 (165 – 180) | 0.496 | 0.086 |
| Weight (kg) | 90 (80 – 110) | 95 (80 – 110) | 88 (80 – 100) | 0.070 | 85 (75 – 100) | 80 (70 – 94) | 90 (75 – 104) | 0.156 | 0.015 |
| Body-Mass Index (BMI) (kg/m^2^) | 29.3 (26.2 – 34.2) | 30.1 (26.8 – 36.6) | 28.3 (16.0 – 32.9) | 0.253 | 28.6 (24.7 – 34.5) | 26.9 (24.0 – 31.9) | 29.4 (25.0 – 35.5) | 0.104 | 0.031 |
| Sequential Organ Failure Assessment (SOFA) | 9 (8 – 12) | 10 (8 – 12) | 9 (8 – 11) | 0.789 | 13 (10 – 15) | 15 (12 – 16) | 12 (9 – 15) | 0.051 | <0.001 |
| Murray Score | 3.3 (3.3 – 3.7) | 3.3 (3.1 – 3.6) | 3.3 (3.3 – 3.7) | 0.428 | 3.3 (3.3 – 3.7) | 3.3 (3 – 3.7) | 3.3 (3.3 – 3.7) | 0.153 | 0.855 |
| Pulmonary compliance before V-V ECMO (mL/mbar) | 26 (19 – 33) | 26 (20 – 35) | 26 (19 – 33) | 0.658 | 28 (22 – 36) | 28 (22 – 34) | 29 (21 – 36) | 0.585 | 0.704 |
| Days from intubation  to V-V ECMO | 3 (1 – 8) | 3 (1 – 8) | 3 (1 – 8) | 0.946 | 1 (0 – 3) | 2 (0 – 9) | 1 (0 – 2) | 0.108 | 0.766 |
| Days from first symptoms to intubation | 7 (2 – 12) | 9 (2 – 15) | 6 (2 – 11) | 0.106 | 1 (0 – 4) | 1 (0 – 4) | 1 (0 – 4) | 0.861 | <0.001 |
| Days from first symptoms to V-V ECMO | 12 (7 – 20) | 15 (8 – 20) | 11 (7 – 19) | 0.187 | 3 (1 – 9) | 7 (3 – 10) | 3 (1 – 7) | 0.025 | 0.001 |
| COVID-19 Variants | | | | 0.745 |  | | | | |
| Alpha Variant, n (%) | 60 (41) | 21 (44) | 39 (39) |  |  |  |  |  |  |
| British Variant, n (%) | 35 (24) | 9 (19) | 26 (26) |  |  |  |  |  |  |
| Delta Variant, n (%) | 50 (34) | 17 (35) | 33 (33) |  |  |  |  |  |  |
| Omikron Variant, n (%) | 2 (1) | 1 (2) | 1 (1) |  |  |  |  |  |  |
| COVID-19 specific therapy | | | | |  |  |  |  |  |
| Glucocorticoids | 137 (93) | 45 (94) | 92 (93) | 0.853 |  |  |  |  |  |
| Convalescent plasma | 37 (25) | 11 (23) | 26 (26) | 0.711 |  |  |  |  |  |
| Monoclonal antibodies | 16 (11) | 5 (10) | 11 (11) | 0.899 |  |  |  |  |  |
| Remdesivir | 31 (21) | 9 (19) | 22 (22) | 0.628 |  |  |  |  |  |
| Litonavir / Ritonavir | 1 (1) | 0 | 1 (1) | 0.483 |  |  |  |  |  |
| Tocilizumab | 10 (7) | 0 | 10 (10) | 0.023 |  |  |  |  |  |
| Hydroxychloroquin | 2 (1) | 0 | 2 (2) | 0.321 |  |  |  |  |  |
| Baricitinib | 9 (6) | 3 (6) | 6 (6) | 0.964 |  |  |  |  |  |
| Laboratory Testing before ECMO | | | | | | | | | |
| Creatinine (mg/dL) | 0.9 (0.7 – 1.5) | 0.8 (0.7 – 1.4) | 0.9 (0.7 – 1.5) | 0.562 | 1.5 (0.8 – 2.5) | 2.5 (1.1 – 3.6) | 1.4 (0.7 – 2.2) | 0.069 | 0.001 |
| Blood Urea (mg/dL) | 57 (37 – 82) | 56 (40 – 83) | 57 (35 – 83) | 0.665 | 64 (40 – 101) | 94 (37 – 141) | 58 (40 – 82) | 0.139 | 0.090 |
| Creatine kinase (U/L) | 187 (60 – 446) | 147 (59 – 491) | 197 (65 – 440) | 0.996 | 350 (151 – 1162) | 800 (150 – 2626) | 283 (151 – 864) | 0.136 | 0.011 |
| LDH (U/L) | 443 (313 – 648) | 501 (338 – 663) | 431 (312 – 651) | 0.362 | 577 (320 – 839) | 620 (459 – 1169) | 550 (302 – 812) | 0.184 | 0.081 |
| ASAT (U/L) | 56 (41 – 89) | 51 (36 – 82) | 63 (41 – 95) | 0.293 | 87 (45 – 1419) | 81 (42 – 238) | 87 (45 – 139) | 0.807 | 0.093 |
| ALAT(U/L) | 54 (35 – 79) | 53 (33 – 75) | 55 (36 – 87) | 0.410 | 41 (24 – 66) | 31 (24 – 56) | 46 (25 – 67) | 0.301 | 0.031 |
| ALP (U/L) | 99 (72 – 155) | 96 (66 – 147) | 100 (72 – 174) | 0.392 | 88 (70 – 151) | 131 (72 – 190) | 85 (69 – 123) | 0.083 | 0.185 |
| Cholinesterase (U/L) | 4062 (3124 – 4945) | 4012 (3273 – 4661) | 4155 (3050 – 5036) | 0.592 | 5534 (3437 – 7277) | 3637 (2098 – 6741) | 5655 (3832 – 7590) | 0.165 | 0.880 |
| Bilirubin (mg/dL) | 0.6 (0.4 – 1) | 0.7 (0.5 – 1.0) | 0.6 (0.4 – 1.0) | 0.448 | 0.7 (0.4 – 1.3) | 1.2 (0.6 – 3.2) | 0.6 (0.4 – 1.1) | 0.018 | 0.019 |
| Albumin (g/L) | 25 (23 – 28) | 25 (22 – 26) | 26 (23 – 29) | 0.374 | 19 (16 – 23) | 20 (25 – 25) | 19 (16 – 22) | 0.877 | 0.008 |
| CRP (mg/L) | 124 (33 – 231) | 164 (39 – 295) | 112 (31 – 209) | 0.068 | 182 (98 – 286) | 130 (44 – 287) | 200 (114 – 283) | 0.257 | 0.812 |
| Procalcitonin (ng/mL) | 0.6 (0.24 – 1.68) | 0.7 (0.3 – 2.0) | 0.6 (0.2 – 1.1) | 0.622 | 2.8 (1.0 – 11.3) | 4 (1.6 – 11.9) | 2.2 (0.9 – 9) | 0.203 | <0.001 |
| aPTT (%) | 88 (75 – 100) | 91 (75 – 100) | 87 (75 – 99) | 0.803 | 91 (75 – 99) | 90 (65 – 100) | 91 (76 – 99) | 0.871 | 0.878 |
| INR | 1.1 (1.0 – 1.2) | 1.1 (1.0 – 1.2) | 1.1 (1.0 – 1.2) | 0.112 | 1.0 (1.0 – 1.2) | 1.1 (1.0 – 1.2) | 1.0 (1.0 – 1.2) | 0.949 | 0.945 |
| Fibrinogen (mg/dL) | 595 (467 – 692) | 616 (488 – 100) | 578 (442 – 690) | 0.183 | 466 (381 – 583) | 466 (421 – 554) | 461 (361 – 619) | 0.789 | 0.011 |
| D-Dimers (mg/L) | 4.0 (2.0 – 9.8) | 4.0 (2.0 – 9.0) | 4.0 (2.0 – 12.0) | 0.597 | 5.0 (3.0 – 9.0) | 5 (4 – 13) | 5 (2 – 9) | 0.340 | 0.340 |
| Antithrombin III (%) | 77 (65 – 89) | 76 (62 – 87) | 78 (66 – 89) | 0.468 | 71 (55 – 82) | 78 (54 – 83) | 70 (55 – 81) | 0.427 | 0.706 |
| sIL2-R (U/mL) | 1614 (1067 – 2475) | 1596 (1065 – 2412) | 1691 (1067 – 2605) | 0.895 | 2186 (1617 – 4341) | 3713 (1763 – 4573) | 2128 (1381 – 3402) | 0.170 | 0.004 |
| TNF (pg/mL) | 16.0 (12.0 – 24.5) | 16.0 (13.0 – 22.0) | 16.0 (12.0 – 26.5) | 0.676 | 24.0 (14.0 – 43.0) | 30.0 (17.0 – 42.5) | 22.0 (14.0 – 44.5) | 0.443 | 0.010 |
| IL-6 (pg/mL) | 106 (48.3 – 664.5) | 93 (29 – 442) | 128 (54 – 739) | 0.114 | 723 (111 – 2567) | 765 (194 – 1707) | 685 (93 – 2897) | 0.746 | 0.002 |
| IL-8 (ng/L) | 57 (35 – 94) | 60 (41 – 117) | 57 (27 – 90) | 0.333 | 149 (67 – 471) | 158 (127 – 433) | 133 (58 – 487) | 0.310 | <0.001 |
| Platelets (1/nL) | 275 (195 – 377) | 281 (215 – 381) | 266 (192 – 378) | 0.621 | 164 (120 – 208) | 166 (106 – 211) | 164 (122 – 208) | 0.818 | <0.001 |
| White blood cells (1/nL) | 11.7 (8.4 – 16.4) | 13 (8.2 – 16.8) | 11.4 (8.5 – 15.8) | 0.385 | 11.2 (8.1 – 16.8) | 8.8 (7.2 – 10.9) | 12 (8.3 – 17.0) | 0.054 | 0.054 |
| Clinical Complications during Therapy | | | | | | | | | |
| AKI | 89 (61) | 43 (48) | 46 (79) | <0.001 | 53 (74) | 33 (64) | 20 (100) | 0.002 | 0.057 |
| AKI before ECMO | 23 (16) | 9 (10) | 14 (24) | 0.022 | 34 (47) | 18 (35) | 16 (80) | 0.001 | <0.001 |
| AKI during ECMO | 66 (45) | 34 (38) | 32 (55) | 0.043 | 19 (26) | 15 (29) | 4 (20) | 0.466 | 0.008 |
| Dialysis | 41 (28) | 12 (11) | 29 (50) | <0.001 | 39 (54) | 22 (42) | 17 (85) | 0.001 | <0.001 |
| Dialysis before ECMO | 4 (3) | 0 | 4 (7) | 0.012 | 13 (18) | 5 (10) | 8 (40) | 0.003 | <0.001 |
| Pulmonary Embolism  (n of CTPA=134) | 51 (38) | 31 (40) | 20 (35) | 0.636 | 5 (8) | 4 (9) | 1 (6) | 0.725 | <0.001 |
| ICB (n CCT=126) | 20 (16) | 6 (8) | 14 (26) | 0.006 | 7 (12) | 2 (5) | 5 (31) | 0.006 | 0.485 |
| Stroke | 5 (4) | 1 (1) | 4 (8) | 0.084 | 4 (7) | 2 (5) | 2 (13) | 0.299 | 0.421 |
| Major Bleeding | 45 (31) | 25 (28) | 20 (34) | 0.411 | 10 (14) | 4 (8) | 6 (32) | 0.012 | 0.010 |
| Proven SSC | 8 (5) | 1 (1) | 7 (12) | 0.004 | 0 (0) | 0 (0) | 0 (0) | - | 0.004 |
| Suspected SSC | 51 (35) | 15 (17) | 36 (62) | <0.001 | 0 (0) | 0 (0) | 0 (0) | - | <0.001 |

Table S 3: Patient characteristics, laboratory testing and specific therapy in patients developing Aspergillosis. Multivariate Analysis. Continuous variables are shown as median and IQR 25^th^- 75^th^. Categorized variables are shown as the number and percentage of group´s size.

Abbreviations: V-V ECMO = Veno-venous extracorporeal membrane oxygenation, LDH = Lactate dehydrogenase, ASAT = Aspartate Aminotransferase, ALAT = Alanine Aminotransferase, ALP = Alkaline phosphatase, CRP = C-reactive protein, aPPT = activated Partial Thromboplastin Time, INR = international normalized ratio, sIL2-R = soluble Interleukin-2 Receptor, TNF = Tumor necrosis factor, IL-6 = interleukin 6, IL-8 = interleukin 8, AKI = acute kidney injury, CTPA = Computed Tomography Pulmonary Angiogram, ICB = intracranial bleeding, CCT = Cranial Computed Tomography, SSC = secondary sclerosing cholangitis

| Study | Patients | Onset of VAP | First Episode | Further Episodes |
| --- | --- | --- | --- | --- |
| Luyt (9) | COVID-19  V-V ECMO (n=50), VAP (n=43) | 10 (8–16) days after intubation | Klebsiella aerogenes 26% Enterobacter cloacae 7%  Pseudomonas aeruginosa 37%  Staphylococcus aureus 7%  Pneumococcus 7% | Pseudomonas aeruginosa 56%  Enterobacteriaceae 47% |
| Rouzé (10) | COVID-19, Mechanical Ventilation  (n=568, 55 with bacterial identification) | Not reported | Staphylococcus aureus 25.4%  Pneumococcus 21.8%  Pseudomonas aeruginosa 10.9%  Haemophilus influenzae 9.1%  Klebsiella spp. 3.6% |  |
| Rouzé (10) | Influenza, Mechanical Ventilation  (n=482, 162 with bacterial identification) | Not reported | Staphylococcus aureus 31.5%  Pneumococcus 32.1%  Pseudomonas aeruginosa 6.2%  Haemophilus influenzae 11.1%  Klebsiella spp. 2.5% |  |
| Annual Epidemiological report 2017 (25) | ICU Patients  (n=5069 in Germany) | Not reported | Pseudomonas aeruginosa 16.1%  Staphylococcus aureus 19.8%  Klebsiella spp. 18.2%  Escherichia coli 16.3%  Enterobacter spp. 9.4% |  |
| Ayzac (22)  PROSEVA Study | ARDS  (n=93) | 12 (7-19) days after intubation | Pseudomonas aeruginosa 21%  Enterobacteriaceae 10%  Escherichia coli 9%  Klebsiella spp. 4%  Staphylococcus aureus 12%  Pneumococcus 3% |  |
| Markowicz (23) | ARDS and VAP  (n=49) | 11.7 ± 11.9  days after intubation | Pseudomonas aeruginosa 31%  Acinetobacter baumanii 10%  Enterobacteriaceae 13.5%  Staphylococcus aureus 21% |  |
| Markowicz (23) | VAP without ARDS  (n=173) | 11.7 ± 11.9  days after intubation | Pseudomonas aeruginosa 26%  Acinetobacter baumanii 6%  Enterobacteriaceae 21%  Staphylococcus aureus 33%  MRSA 18% |  |
| Bal (27) | Influenza on ICU | Not reported | Staphylococcus aureus 20%  Strept. pneumoniae 15.6%  Strept. Pyogenes 4.4%  Haemophilus influenzae 11.1% |  |
| Andersen (6) | COVID-19 (n=50) | Not reported | Staphylococcus aureus 6%  Pseudomonas aeruginosa 18%  Stenotrophomonas 8%  Acinetobacter baumanii 6%  Klebsiella spp. 6%  Enterobacter spp. 6% |  |
| De Hesselle (7) | COVID-19 (n=840), Mechanical Ventilation (n=429), V-V ECMO (n=114/840) | Not reported | Not reported |  |

Table S 4: Literature data describing bacterial pathogens causing ventilator associated pneumonia (VAP)
Abbreviations: V-V ECMO = veno-venous extracorporeal oxygenation, ICU = intensive care unit, ARDS = adult respiratory distress syndrome, MRSA = methicillin resistant staphylococcus aureus, Strept. = Streptococcus

|  | COVID-19 | | | | Influenza | | | |  |
| --- | --- | --- | --- | --- | --- | --- | --- | --- | --- |
| Variable | All  (n=147) | Survivor  (n=89) | Non-Survivor  (n=58) | p-value | All (n=72) | Survivor  (n=52) | Non-Survivor  (n= 20) | p-value | p-value  (COVID-19 vs Influenza) |
| Pulmonary bacterial superinfection,  n (% of all) | 89 (61) | 55 (62) | 34 (59) | 0.700 | 25 (35) | 20 (39) | 5 (25) | 0.283 | **<0.001** |
| Initial bacterial pathogen resistant to antibiotics, n (%) | 28 (31) | 13 (15) | 15 (26) | **0.043** | 6 (8) | 6 (12) | 0 (0) | 0.160 | 0.510 |
| Detection of resistant bacterial pathogens / other than initial, n (%) | 6 (7) | 2 (5) | 4 (3) | 0.754 | 0 (0) | 0 (0) | 0 (0) | - | 0.082 |
| Development of resistance to antibiotics, n (%) | 18* (20) | 11 (12) | 7 (12) | 0.958 | 1 (1) | 1 (2) | 0 (0) | 0.532 | **0.007** |
| Days from first bacterial pathogen detection to resistance detection of initial pathogens | 6 (3 – 22) | 5 (3 – 25) | 8 (4 – 21) | 0.994 | 4 | 4 | - | - | - |
| Days from first symptoms to first pulmonary bacterial pathogen detection | 18 (11 – 32) | 18 (9 – 38) | 19 (13 – 28) | 0.979 | 4 (1– 30) | 9 (1 – 35) | 2 (-1 – 8) | 0.169 | **0.005** |
| Days from intubation to first pulmonary bacterial pathogen detection | 8 (2 – 19) | 8 (2 – 28) | 8 (2 – 15) | 0.362 | 1 (0 – 27) | 4 (0 – 34) | 0 (-1 – 1) | 0.071 | 0.051 |
| Days from ECMO to first pulmonary bacterial pathogen detection | 2 (1 – 15) | 3 (1 – 23) | 1 (0 – 8) | 0.116 | -1 (-3 – 22) | 0 (-3 – 27) | -1 (-7 – 1) | 0.272 | **0.019** |

Table S 5: Pulmonary bacterial infection with regards to antibiotic-resistances. *2 Patients developed more than one pathogen resistant to antibiotics. Continuous variables are shown as median and IQR 25^th^- 75^th^. Categorized variables are shown as the number and percentage of group´s size.
Abbreviations: ECMO = extracorporeal membrane oxygenation

|  | COVID-19 | | | | Influenza | | | |  |
| --- | --- | --- | --- | --- | --- | --- | --- | --- | --- |
|  | All  (n=147) | Survivor  (n=89) | Non-Survivor  (n=58) | p-value | All (n=72)* | Survivor  (n=52) | Non-Survivor  (n= 20) | p-value | p-value  (COVID-19 vs Influenza) |
| Ever positive blood culture | 78 (52) | 43 (48) | 35 (60) | 0.153 | 13 (18) | 8 (15) | 5 (25) | 0.342 | <0.001 |
| Subset of detected spectrum in positive blood culture in number of patients (in % of cohort) | | | | | | | | | |
| Ever positive blood culture excluding CNS-detection (in % of subset of cohort) | 35 (23) | 17 (19) | 18 (51) | 0.115 | 4 (6) | 3 (6) | 1 (5) | 1.000 | <0.001 |
| Ever positive blood culture with CNS in at least 1 culture during ICU stay (in % of subset of cohort) | 53 (36) | 30 (33) | 23 (26) | 0.486 | 5 (7) | 4 (8) | 1 (5) | 1.000 | <0.001 |
| Ever positive blood culture classified as probable infection with CNS in at least 1 blood culture during ICU stay | 15 (10) | 10 (11) | 5 (6) | 0.782 | 5 (7) | 0 (0) | 1 (5) | 1.000 | 0.001 |
| Ever Enterococci in blood culture | 21 (27) | 12 (28) | 9 (26) | 0.730 | 1 (1) | 1 (2) | 0 (0) | 0.439 | 0.134 |
| Ever Pseudomonas aeruginosa in blood culture | 2 (3) | 1 (2) | 1 (3) | 0.759 | 0 (0) | 0 (0) | 0 (0) | - | 0.559 |
| Ever Staphylococcus aureus in blood culture | 5 (6) | 1 (2) | 4 (11) | 0.59 | 2* (3) | 1(2) | 1 (5) | 0.649 | 0.261 |
| Ever Klebsiella pneumoniae in blood culture | 2 (3) | 2 (5) | 0 (0) | 0.250 | 0 (0) | 0 (0) | 0 (0) | - | 0.559 |
| Ever Enterobacter in blood culture | 1 (1) | 1 (2) | 0 (0) | 0.418 | 0 (0) | 0 (0) | 0 (0) | - | 0.681 |
| Ever Acinetobacter baumanii in blood culture | 1 (1) | 0 (0) | 1 (3) | 0.214 | 0 (0) | 0 (0) | 0 (0) | - | 0.757 |
| Ever Proteus mirabilis in blood culture | 1 (1) | 0 (0) | 1 (3) | 0.214 | 0 (0) | 0 (0) | 0 (0) | - | 0.681 |
| Ever Serratia marcescens in blood culture | 1 (1) | 1 (2) | 0 (0) | 0.418 | 0 (0) | 0 (0) | 0 (0) | - | 0.681 |
| Ever Candida spp. in blood culture | 5 (6) | 0 (0) | 5 (14) | 0.005 | 2* (3) | 2 (4) | 0 (0) | 0.224 | 0.217 |
| Ever Escherichia coli in blood culture | 1 (1) | 1 (2) | 0 (0) | 0.418 | 0 (0) | 0 (0) | 0 (0) | - | 0.681 |

Table S 6: Pathogen detected bloodstream infections in COVID-19 and Influenza patients.
Abbreviations: CNS = coagulase negative staphylococci, ICU = intensive care unit

| Aspergillus | | | | | | | | | |
| --- | --- | --- | --- | --- | --- | --- | --- | --- | --- |
|  | COVID-19 | | | | Influenza | | | |  |
|  | All  (n=147) | Survivor  (n=89) | Non-Survivor  (n=58) | p-value | All (n=72) | Survivor  (n=52) | Non-Survivor  (n= 20) | p-value | p-value  (COVID-19 vs Influenza) |
| Positive culture, n (%) | 26 (18) | 15 (17) | 11 (19) | 0.743 | 8 (11) | 3 (6) | 5 (25) | 0.020 | 0.207 |
| Days from first symptoms to positive culture from respiratory material (median | 20 (12 – 30) | 18 (12 – 25) | 23 (12 – 35) | 0.610 | 11 (9 – 30) | 10 | 11 (10 – 27) | 0.786 | 0.253 |
| Days from intubation to positive culture | 9 (5 – 21) | 8 (5 – 20) | 9 (4 – 24) | 0.799 | 10 (3 – 26) | 10 | 10 (4 – 22) | 0.786 | 0.796 |
| Days from V-V ECMO to positive culture | 6 (2 – 10) | 6 (2 – 15) | 5 (0 – 10) | 0.646 | 6 (0 – 26) | 6 | 6 (1 – 22) | 1 | 0.858 |
| Galactomannan Index >1 BAL,  n (%) | 29 (20) | 14 (16) | 15 (26) | 0.083 | 8 (11) | 4 (8) | 4 (20) | 0.100 | 0.492 |
| Days from Intubation to maximal Galactomannan Index BAL | 18 (9 – 36) | 16 (9 – 32) | 19 (9 – 38) | 0.683 | 11 (3 – 17) | 13 (5 – 18) | 5 (1 - 15) | 0.135 | <0.001 |
| Galactomannan Index >0.5 serum, n (%) | 16 (11%) | 7 (8) | 9 (16) | 0.066 | 5 (7) | 1 (2) | 4 (20) | 0.024 | 0.821 |
| Days from intubation to maximal Galactomannan Index Serum | 17 (8 – 30) | 20 (8 – 34) | 15 (8 – 27) | 0.262 | 13 (4 – 20) | 17 (13 – 23) | 5 (1 – 10) | <0.001 | 0.032 |
| Probable aspergillosis n (%) | 48 (33%) | 25 (28%) | 23 (40%) | 0.144 | 16 (22) | 8 (15) | 8 (40) | 0.024 | 0.111 |
| Days from first symptoms to aspergillosis diagnosis | 23 (16 – 42) | 20 (12 – 34) | 26 (18 – 42) | 0.115 | 11 (8 – 14) | 14 (9 – 39) | 9 (4 – 11) | 0.065 | 0.001 |
| Days from intubation to aspergillosis diagnosis | 15 (8 – 31) | 13 (5 – 26) | 21 (9 – 34) | 0.252 | 8 (2 – 13) | 12 (9 – 38) | 4 (1 – 7) | 0.005 | 0.026 |
| Days from V-V ECMO to aspergillosis diagnosis | 9 (2 – 24) | 10 (2 – 22) | 8 (2 – 24) | 0.886 | 3 (1 – 9) | 8 (1 – 31) | 2 (0 – 5) | 0.161 | 0.055 |
| Days from antimycotic treatment to aspergillosis diagnosis | 7 (1 – 19) | 5 (0 – 20) | 7 (1 – 19) | 0.843 | 0 (-1 – 1) | 0 (-1 – 16) | 0 (-1 – 0) | 0.445 | 0.003 |
| Preemptive Antifungal therapy, n (%) | 127 (86%) | 75 (85%) | 52 (88%) | 0.661 | 49 (68) | 33 (64) | 16 (80) | 0.178 | 0.001 |
| Aspergillosis despite preemptive therapy, n (%) | 48 (33) | 25 (28) | 23 (40) | 0.164 | 13 (18) | 6 (12) | 7 (35) | 0.057 | 0.159 |
| Zygomycetes, n (%) | 3 (2%) | 1 (2%) | 2 (2%) | 0.826 | 1 (1) | 1 (2) | 0 (0) | 0.343 | 0.095 |
| Died from Aspergillosis, n (%) | 6 (4) |  | 6 (10% of all, 25% of subgroup) |  | 1 (1) |  | 1 (5) |  |  |

Table S 7: Aspergillus diagnostic in COVID-19 and influenza patients on veno-venous extracorporeal membrane oxygenation (V-V ECMO).
Continuous variables are shown as median and IQR 25^th^- 75^th^. Categorized variables are shown as number and percentage of group’s size.
Abbreviations: BALF = Bronchoalveolar Lavage Fluid

| Cytomegalovirus | | | | | | | | | |
| --- | --- | --- | --- | --- | --- | --- | --- | --- | --- |
|  | COVID-19 | | | | Influenza | | | |  |
|  | All  (n=147) | Survivor  (n=89) | Non-Survivor  (n=58) | p-value | All (n=72) | Survivor  (n=52) | Non-Survivor  (n= 20) | p-value | p-value  (COVID-19 vs Influenza) |
| Systemic CMV reactivation, n (%) | 26 (18) | 14 (16%) | 12 (21%) | 0.441 | 2 (3) | 1 (2) | 1 (5) | 0.596 | 0.002 |
| Days from first symptoms to systemic CMV reactivation | 54 (40 – 62) | 43 (32 – 62) | 60 (53 – 68) | 0.118 | 28 | 44 | 11 | 1 | 0.222 |
| Days from Intubation to systemic CMV reactivation | 43 (24 – 57) | 29 (23 – 57) | 48 (42 – 58) | 0.085 | 27 | 42 | 11 | 1 | 0.259 |
| Days from V-V ECMO to systemic CMV reactivation | 37 (22 – 49) | 26 (22 – 49) | 38 (36 – 50) | 0.347 | 23 | 37 | 9 | 1 | 0.296 |
| Pulmonary CMV reactivation, n (%) | 28 (19%) | 16 (18%) | 12 (21%) | 0.682 | 3 (4) | 2 (4) | 1 (5) | 0.835 | 0.009 |
| Days from first symptoms to pulmonary CMV reactivation | 51 (27 – 45) | 49 (43 – 57) | 54 (43 – 65) | 0.569 | 34 | 43 | 19 | 1 | 0.122 |
| Days from Intubation to pulmonary CMV reactivation | 39 (33 – 54) | 39 (32 – 49) | 47 (28 – 56) | 0.589 | 29 | 39 | 19 | 1 | 0.285 |
| Days from V-V ECMO to pulmonary CMV reactivation | 37 (27 – 45) | 32 (26 – 44) | 36 (23 – 42) | 0.664 | 27 | 36 | 17 | 1 | 0.462 |
| Herpes Simplex Virus | | | | | | | | | |
|  | COVID-19 | | | | Influenza | | | |  |
| Pulmonary HSV reactivation, n (%) | 72 (49%) | 43 (48%) | 29 (50%) | 0.841 | 19 (26) | 14 (27) | 5 (25) | 0.949 | 0.005 |
| Days from first symptoms to pulmonary HSV reactivation | 27 (20 – 35) | 27 (21 – 38) | 27 (20 – 33) | 0.807 | 16 (13 – 25) | 15 (13 – 20) | 9 (13 – 27) | 0.612 | 0.001 |
| Days from Intubation to pulmonary HSV reactivation | 18 (11 – 27) | 21 (11 – 32) | 18 (10 – 24) | 0.245 | 15 (11 – 20) | 14 (11 – 20) | 18 (12 – 22) | 0.612 | 0.275 |
| Days from V-V ECMO to pulmonary HSV reactivation | 10 (5 – 22) | 14 (5 – 25) | 9 (6 – 19) | 0.236 | 13 (9 – 18) | 11 (9 – 16) | 17 (7 – 21) | 0.497 | 0.618 |

Table S 8: Viral reactivations in COVID-19 and influenza patients regarding systemic and pulmonary cytomegalovirus (CMV) and pulmonary reactivation of Herpes Simplex Virus (HSV) requiring veno-venous extracorporeal membrane oxygenation (V-V ECMO).
Continuous variables are shown as median and IQR 25th- 75th. Categorized variables are shown as number and percentage of group’s size.

|  | | Antifungal therapy near ECMO-Initiation | | | Preemptive antifungal therapy | | |
| --- | --- | --- | --- | --- | --- | --- | --- |
| antifungal drug during ICU therapy | | Initial antifungal drug for CAPA/IAPA diagnosed before ECMO-initiation | Initial antifungal therapy before/ within 5 days of ECMO without CAPA/ IAPA | Initial antifungal drug later than 5 days on ECMO | No sufficient duration * | Break-through aspergillosis** | Adequate preemptive therapy and no aspergillosis *** |
| COVID-19 (n=147)† | | | | | | | |
|  | n=127 (86%) | n=4 (3%) | n=98 (67%) | n=25 (17%) | n=13 (9%) | n=22 (15%) | n=79 (54%) |
| Anidulafungin | 1 (1%) | 0 | 1 (1%) | 0 | 0 | 0 | 1(1%) |
| Caspofungin | 110 (75%) | 3 (2%) | 85 (58%) | 22 (15%) | 12 (8%) | 21 (14%) | 72(49%) |
| Caspofungin Isavucon-azole | 1 (1%) | 0 | 1 (1%) | 0 | 0 | 0 | 0 |
| Isavucon-azole | 5 (3%) | 0 | 3 (2%) | 2 (1%) | 1(1%) | 1 (1%) | 1 (1%) |
| Posaconazole | 1 (1%) | 0 | 1 (1%) | 0 | 0 | 0 | 1(1%) |
| Voriconazole | 9 (6%) | 1(1%) | 7 (5%) | 1 (1%) | 0 | 0 | 4(3%) |
| Influenza (n=72) ‡ | | | | | | | |
|  | n=49 (68%) | n=3 (4%) | n=25 (35%) | n=21 (29%) | N=2 (3%) | N=1 (1%) | n=36 (50%) |
| Amphotericin B * | 1 (1%) | 1 (1%) | 0 | 0 | 0 | 0 | 0 |
| Caspofungin | 9 (13%) | 1 (1%) | 5 (7%) | 3 (4%) | 1 (1%) | 0 | 6 (8%) |
| Posaconazole | 2 (3%) | 0 | 1 (1%) | 1 (1%) | 0 | 1(1%) | 1 (1%) |
| Voriconazole | 37 (51%) | 1 (1%) | 19 (26%) | 17 (24%) | 1(1%) | 0 | 29 (40%) |

Table S 9: Antifungal therapy and deterioration with need for veno-venous extracorporeal membrane oxygenation (V-V ECMO). * First antimycotic drug in patients developing Aspergillosis within 7 days after first administration of Antimycotic; ** First antimycotic drug in patients developing Aspergillosis after more than 7 days after first administration of Antimycotic Drug, *** First antimycotic drug in patients developing no Aspergillosis
†Amphotericin B was not administered to any COVID-19 patients., ‡ Neither Anidulafungin nor Isavuconazole (alone or with Caspofungin) was administered in Influenza patients.
Abbreviations: ICU = intensive care unit, CAPA = COVID-19 Associated Pulmonary Aspergillosis, IAPA = Influenza Associated Pulmonary Aspergillosis

Figure S1: Comparison of clinical courses of patients on veno-venous extracorporeal membrane oxygenation (V-V ECMO) because of COVID-19 (Figure S1A) or Influenza (Figure S1 B) and additional superinfections. Survival rates in each subgroup are calculated in % of each group.
Abbreviations: V-V ECMO = Veno-venous extracorporeal membrane oxygenation, HSV = Herpes Simplex Virus, CMV = Cytomegalovirus

Figure S1 A: Clinical course of patients on V-V ECMO because of COVID-19 and superinfection. Reference time is onset of first symptoms. Survival rates display survival in subgroups. Time is displayed as Median and IQR 25^th^- 75^th^.*1 COVID-19 patients did not fulfill criteria for Ventilator associated pneumonia (VAP) because of ventilation time being <48 hours before diagnosis of pulmonary bacterial superinfection.

Figure S 1 B: Clinical course of patients on V-V ECMO because of Influenza and superinfection. Reference time is onset of first symptoms. Survival rates display survival in subgroups. Time is displayed as Median and IQR 25^th^- 75^th^. *11 Influenza patients did not fulfill criteria for Ventilator associated pneumonia (VAP) because of ventilation time being <48 hours before diagnosis of pulmonary bacterial superinfection.

Figure S2: Baseline characteristics regarding inflammatory markers being significantly higher in Influenza patients than in COVID-19- Patients: CRP (C-reactive Protein) p<0.002; sIL-2R (soluble interleukin 2 receptor), TNF-α (tumor necrosis factor-α), as well as Interleukin-6 (IL-6) and Interleukin-8 (IL-8) p<0.001. Outliers are not displayed.

Figure S3: prevalence of bacterial pathogens in COVID-19 and Influenza patients. Values display percentage of subgroup with/without detected pulmonary pathogens.

Figure S4: Classification of bacterial pathogens in the first episode of pulmonary bacterial superinfections. Values display percentage of all detected bacterial pathogens in cohort during the intensive care unit stay. (*other: Serratia rubidaea, Delftia acidovorans, Raoultella planticola, Moraxella spp., Morganella spp, Acinetobacter baumanii, Streptococcus pneumoniae; each =1%).
Abbreviations: E. coli = Escherichia coli

**ECMO Management**

UKR-ECMO center follows the Extracorporeal Life Support Organization (ELSO) https://www.elso.org/Resources/Guidelines.aspx guidelines for ECMO indications, equipment setup and ECMO management parameters. Our ECMO team closely monitors the ECMO circuit for evidence of thrombosis (visual inspection, oxygenator blood gas analysis, oxygenator pressures) and hemolysis (daily plasma cell free hemoglobin levels and daily complete blood counts). Anticoagulation was managed with unfractionated heparin, usually targeting an activated partial thromboplastin time (aPTT) of 50-60 seconds. In cases of heparin-induced thrombocytopenia, argatroban was used as an alternative anticoagulant.

STROBE Statement:
Abbreviations: n.a. = not applicable

|  | Item No | Recommendation |  |  | Page |
| --- | --- | --- | --- | --- | --- |
| **Title and abstract** | 1 | (*a*) Indicate the study’s design with a commonly used term in the title or the abstract |  |  | 2 |
|  |  | (*b*) Provide in the abstract an informative and balanced summary of what was done and what was found |  |  | 2 |
| Introduction | | |  |  |  |
| Background/rationale | 2 | Explain the scientific background and rationale for the investigation being reported |  |  | 4 |
| Objectives | 3 | State specific objectives, including any prespecified hypotheses |  |  | 4 |
| Methods | | |  |  |  |
| Study design | 4 | Present key elements of study design early in the paper |  |  | 5 |
| Setting | 5 | Describe the setting, locations, and relevant dates, including periods of recruitment, exposure, follow-up, and data collection |  |  | 5 |
| Participants | 6 | (*a*) Give the eligibility criteria, and the sources and methods of selection of participants. Describe methods of follow-up |  |  | 5 |
|  |  | (*b*) For matched studies, give matching criteria and number of exposed and unexposed |  |  | n.a. |
| Variables | 7 | Clearly define all outcomes, exposures, predictors, potential confounders, and effect modifiers. Give diagnostic criteria, if applicable |  |  | 5-7 |
| Data sources/ measurement | 8* | For each variable of interest, give sources of data and details of methods of assessment (measurement). Describe comparability of assessment methods if there is more than one group |  |  | 5-8 |
| Bias | 9 | Describe any efforts to address potential sources of bias |  |  | 8 |
| Study size | 10 | Explain how the study size was arrived at |  |  | 5 |
| Quantitative variables | 11 | Explain how quantitative variables were handled in the analyses. If applicable, describe which groupings were chosen and why |  |  | 8 |
| Statistical methods | 12 | (*a*) Describe all statistical methods, including those used to control for confounding |  |  | 8 |
|  |  | (*b*) Describe any methods used to examine subgroups and interactions |  |  | 8 |
|  |  | (*c*) Explain how missing data were addressed |  |  | 8 |
|  |  | (*d*) If applicable, explain how loss to follow-up was addressed |  |  | n.a. |
|  |  | (*e*) Describe any sensitivity analyses |  |  | 8 |
| Results | | |  |  |  |
| Participants | 13* | (a) Report numbers of individuals at each stage of study—eg numbers potentially eligible, examined for eligibility, confirmed eligible, included in the study, completing follow-up, and analysed |  |  | n.a. |
|  |  | (b) Give reasons for non-participation at each stage |  |  | n.a. |
|  |  | (c) Consider use of a flow diagram |  |  | 23 |
| Descriptive data | 14* | (a) Give characteristics of study participants (eg demographic, clinical, social) and information on exposures and potential confounders |  |  | 9 |
|  |  | (b) Indicate number of participants with missing data for each variable of interest |  |  | n.a. |
|  |  | (c) Summarise follow-up time (eg, average and total amount) |  |  | n.a. |
| Outcome data | 15* | Report numbers of outcome events or summary measures over time |  |  | 12-23 |
| Main results | 16 | (*a*) Give unadjusted estimates and, if applicable, confounder-adjusted estimates and their precision (eg, 95% confidence interval). Make clear which confounders were adjusted for and why they were included |  |  | 12-23 |
|  |  | (*b*) Report category boundaries when continuous variables were categorized |  |  | 12-23 |
|  |  | (*c*) If relevant, consider translating estimates of relative risk into absolute risk for a meaningful time period |  |  | n.a. |
| Other analyses | 17 | Report other analyses done—eg analyses of subgroups and interactions, and sensitivity analyses |  |  | 12-23 |
| Discussion | | |  |  |  |
| Key results | 18 | Summarise key results with reference to study objectives |  |  | 25 |
| Limitations | 19 | Discuss limitations of the study, taking into account sources of potential bias or imprecision. Discuss both direction and magnitude of any potential bias |  |  | 28 |
| Interpretation | 20 | Give a cautious overall interpretation of results considering objectives, limitations, multiplicity of analyses, results from similar studies, and other relevant evidence |  |  | 29 |
| Generalisability | 21 | Discuss the generalisability (external validity) of the study results |  |  | 29 |
| Other information | | |  |  |  |
| Funding | 22 | Give the source of funding and the role of the funders for the present study and, if applicable, for the original study on which the present article is based |  |  | 52 |
